# Recovery of Conformational Continuum From Single-Particle Cryo-EM Images: Optimization of ManifoldEM Informed by Ground Truth

**DOI:** 10.1109/tci.2022.3174801

**Published:** 2022-05-12

**Authors:** Evan Seitz, Francisco Acosta-Reyes, Suvrajit Maji, Peter Schwander, Joachim Frank

**Affiliations:** Department of Biochemistry and Molecular Biophysics, Columbia University Medical Center, New York, NY 10032 USA, and also with the Department of Biological Sciences, Columbia University, New York, NY 10027 USA; Department of Biochemistry and Molecular Biophysics, Columbia University Medical Center, New York, NY 10032 USA; Department of Biochemistry and Molecular Biophysics, Columbia University Medical Center, New York, NY 10032 USA; Department of Physics, University of Wisconsin-Milwaukee, Milwaukee, WI 53211 USA; Department of Biochemistry and Molecular Biophysics, Columbia University Medical Center, New York, NY 10032 USA, and also with the Department of Biological Sciences, Columbia University, New York, NY 10027 USA

**Keywords:** Biomolecules, free-energy landscape, kernel methods, manifold embedding, quantitative biology, single particle cryogenic microscopy (cryo-EM), spectral geometry, unsupervised machine learning

## Abstract

This work is based on the manifold-embedding approach to study biological molecules exhibiting continuous conformational changes. Previous work established a method—now termed ManifoldEM—capable of reconstructing 3D movies and accompanying free-energy landscapes from single-particle cryo-EM images of macromolecules exercising multiple conformational degrees of freedom. While ManifoldEM has proven its viability in several experimental studies, critical limitations and uncertainties have been found throughout its extended development and use. Guided by insights from studies with cryo-EM ground-truth data, simulated from atomic structures undergoing conformational changes, we have built a novel framework, ESPER, able to retrieve the free-energy landscape and respective 3D Coulomb potential maps for all states simulated. As shown by a direct comparison of ground truth vs. recovered maps, and analysis of experimental data from the 80S ribosome and ryanodine receptor, ESPER offers substantial improvements relative to the previous work.

## Graphical Abstract

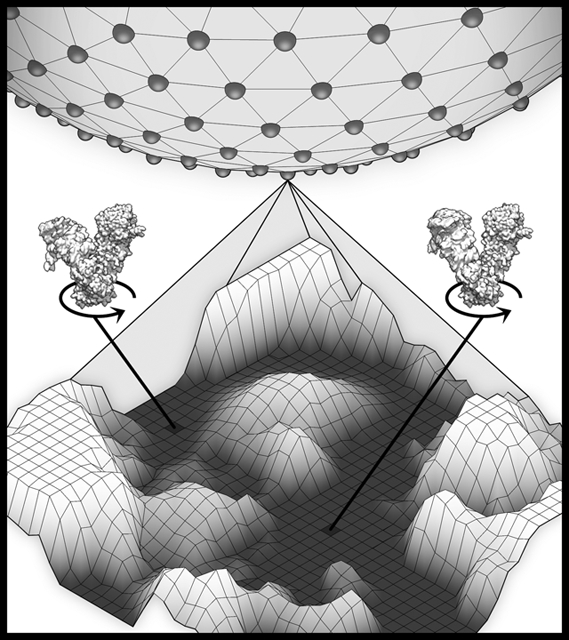

## I. Introduction

MOLECULAR machines—consisting of assemblies of proteins or nucleoproteins—take on a range of unique configurations or *conformational states* as they go through their functional cycles [1]. These states are typically characterized by different spatial constellations of relatively rigid domains, and can be organized in a *state space* according to the continuous motions of each domain along a unique coordinate. Specific sequences of the states in this space form pathways along which the molecular machine may transform. When the number of occurrences of each state is known, the machine’s free-energy landscape can be determined, and a path is singled out along which the machine performs its metabolic function [2].

A number of recent studies [1], [3], [4] were inspired by the realization that it is possible, through the analysis of experimental data, to gain insights into the rules governing a molecular machine’s function. In thermal equilibrium, these machines are constantly buffeted by the random motions of nearby solvent molecules which deform them reversibly as they transition via a series of thermally-driven steps. State-of-the-art single-particle cryo-EM [5]–[7] is now capable of providing large numbers of two-dimensional snapshots (i.e., projections) of a molecular machine undergoing this process. When the number of snapshots is sufficiently large—typically several hundred thousand—they capture virtually the entire range of conformations accessible in thermodynamic equilibrium. By virtue of the Boltzmann statistics, the relative number of sightings in each of these states can be translated into changes of free energy [8], [9]. Thus, under assumption of thermodynamic equilibrium, the machine’s free-energy landscape can be gauged from an experiment. Accurate estimation of this landscape for macromolecular assemblies is of unparalleled importance in modern structural biology.

The way to make use of the data from a single-particle cryo-EM experiment is not easy, however. Ideally, we would wish to compare 3D structures, but only 2D images are accessible experimentally. Each of these 2D images is a *P* pixel projection of the macromolecule, which is assigned an angular viewing direction on the unit sphere *S*^2^. The challenge then is that the relationship among the *N* images requires an analysis of a manifold Ω embedded in a high-dimensional Euclidean space ${\mathbb{R}}^{P}$, which is organized according to both conformational and orientational degrees of freedom. As manifolds are encountered in many domains of mathematics, science and engineering [10], *dimensionality reduction* has been widely pursued and given rise to a number of well-established techniques to analyze large and complex data sets. Representing data points on Ω in terms of leading eigenvalues and eigenvectors gives valuable insights into the manifold’s intrinsic structure, as these relationships have been well studied in the context of spectral geometry [11]. By means of dimensionality reduction, a suitable embedding can be chosen that maps the data points in Ω into a low-dimensional Euclidean space, thus creating the basis for the analysis of the molecule’s conformational spectrum and free-energy landscape.

In the analysis of cryo-EM data, both linear [3], [12]–[18] and nonlinear [1], [19], [20] dimensionality-reduction methodshave been applied, primarily principal component analysis (PCA) [21] and diffusion maps (DM) [22], [23]. Both approaches allow an analysis of the data points in Ω as embedded in ${\mathbb{R}}^{N}$, whose entries are the first *N* eigenvectors of the respective graph. Only a leading subset of these are needed for retrieving the conformational spectrum in good approximation. In the PCA approach, eigenvectors are obtained from the covariance matrix, whereas DM approximates the eigenfunctions of the Laplace-Beltrami operator (LBO) on Ω, sampled at the given data points. Another method, called Laplacian spectral volumes [4], relies on both linear and nonlinear dimensionality reduction. These methods can further be classified based on their type of data input: generating embeddings from either 2D projections straight from a cryo-EM experiment [1], [24], or from 3D density maps reconstructed from those projections [4], [25]–[28]. It is expected that these competing manifold embedding methods should deliver equivalent information when cross-validated, and likewise for alternative techniques, which extend now into work using deep learning [29]–[32].

Here we follow the former strategy, making use of raw 2D projections from single-particle cryo-EM. Images are first grouped by *projection direction* (PD) on *S*^2^ and aligned. In the following we use the term PD for a group of projections with orientations centered on a grid point on *S*^2^ and falling within a given angular aperture width. This width is determined by the stipulation that changes of the image within the aperture due to orientation are small compared to conformational changes. Similarities between images within a PD appear as closeness between corresponding points in the *N*-dimensional space. The geometric structure formed by such an ensemble is a manifold with an intrinsic dimension *n* equal to the number of the system’s independent molecular degrees of freedom. In that manifold, for a given PD, images of molecules captured in random states are arranged—by virtue of their similarities—in the sequence of their continuous conformational motions.

In the following, we use the term *PD-manifold approach* to refer to this strategy, which entails an analysis of the *n*-manifold embedding of each PD, and the combination of resulting representations from all PDs across *S*^2^ to form a consolidated conformational spectrum. Specifically, for each PD independently, an embedding is first formed using nonlinear dimensionality reduction (via diffusion mapping), followed by several applications of nonlinear Laplacian spectral analysis (NLSA) [33] along different coordinates of the embedded space to reconstruct a series of images associated with each degree of freedom in the data set (as seen from that PD). This conformational information is then compiled across all PDs to form an occupancy map and corresponding free-energy landscape, in conjunction with 3D conformational movies. This approach was first introduced by Dashti *et al*. (2014) and is now termed ManifoldEM [24]. Results from previous ManifoldEM studies on biological systems—including the ribosome [1], ryanodine receptor [24], and SARS-CoV-2 spike protein [34]—have proven its viability and its potential to provide new information on the biological function of the molecules.

Since its introduction [24], ManifoldEM has been released to the public through both Matlab [35] and Python [36] distributions, with the latter providing a comprehensive graphics user interface, training manual, and enhanced automation schemes [37]. Throughout these developments [36], the performance of ManifoldEM software and methodology were analyzed extensively by both internal and external testing using several experimental data sets [38]. Some problems and indications of anomalies emerged during these studies [38], but without a comparison to ground truth, their origin could not be traced.

It was the absence of information on what outcome might be expected for a molecular structure undergoing conformational variations that motivated us to analyze the performance of ManifoldEM rigorously with synthetic data [39], [40]. In the course of this detailed heuristic analysis [41] we discovered distinct features of the manifold that enable us to improve the method of analysis and reduce the observed problems. The present account is a condensed version of [41] focusing on seminal results of general interest. Additionally, alternative synthetic data are introduced and analyzed, pseudocode is added detailing three important steps for clarity, and an additional study is conducted on experimental data; altogether, these additions further grant an updated discussion.

We thus introduce a novel methodology (which we will term ESPER: *Embedded subspace partitioning and eigenfunction realignment)* which is able to properly navigate the *n*-dimensional PD-manifold embeddings observed and accurately generate the molecular machine’s free-energy landscape as well as 3D movies depicting its function. Whereas the previous approach [1], [24] aims to reconstruct images via NLSA in an additionally embedded space spanned by one or more conformational motions (CMs), ESPER instead captures each CM directly from the initial embedding while retaining the original cryo-EM raw images. In addition, several novel operations and refinements to the existing PD-manifold approach are introduced, including a previously unaccounted-for high-dimensional eigenbasis transformation that proved essential for correctly recapitulating ground-truth information, as well as identification of the proper 2D subspaces required to adequately capture each CM. We demonstrate that this alternative methodology provides results of significantly improved quality.

## II. Simulation of Cryo-Em Ensembles

We first introduce our framework for the creation of synthetic ground-truth single-particle cryo-EM data sets in the form of 2D projections of 3D density maps arising from a quasi-continuum of atomic structures [39], [40]. In the time since its conception, this synthetic frameworkhas already been used as a performance benchmark by two other groups [29], [42]. To begin, a suitable macromolecule is chosen as a foundational model, defined by structural information available in the form of 3D atomic coordinates from the Protein Data Bank (PDB) [43]. Using this initial PDB structure as a seed, a sequence of states is generated by altering the positions of specific domains of the macromolecule’s structure. To mimic quasi-continuous CMs, we used equispaced rotations of the domains about their hinge-residue axes. The number of these mutually independent CMs defines the intrinsic dimensionality *n* of the system. By exercising these domain motions independently in all combinations, a set of atomic coordinate structures in PDB-format are generated. In sum, this quasi-continuum of states spans the molecular machine’s state space (SS*n*).

For this work, the heat shock protein Hsp90 was chosen due to its illustrative design, exhibiting two arm-like domains connected together in an overarching V-shape which naturally undergo large conformational changes [44]. We initiated our workflow with the fully closed state via entry PDB 2CG9, whose structure was determined at 3.1 Å by X-ray crystallography [45]. Instead of a single conformational motion (arms open to closed, as *in vivo*), we decided to create three easily-identifiable and fully-decoupled domain motions, which we refer to as CM_1_, CM_2_ and CM_3_. Using combinations of these CMs, three synthetic state spaces (SS*n*) were generated, with intrinsic dimensionalities of *n* = 1, 2, 3. In-depth details for these data sets, such as exact atomic descriptions of each state, are provided in Supplementary Material Section A.

Image artifacts and ensemble statistics are also incorporated into these state-space models in four steps, termed data-type I, II, III and IV, with each step designed to move closer to emulating characteristics anticipated in a cryo-EM experiment. Data-type I is given no simulated experimental artifacts or occupancy assignments, which allows us to analytically quantify the trajectories of our simulated conformational changes under ideal settings (Movie 1). In data-type II, we vary the abundance of images (*τ*) per state in each data set and add noise to the images with varying signal-to-noise ratio (SNR), so as to quantify the robustness of this geometry in the presence of noise and statistical coverage. In data-type III, we further apply a contrast transfer function (CTF) with realistic microscopy parameters and random defocus variations (within the typical range expected in the experiment), and add noise to obtain an experimentally-relevant SNR. Finally, data-type IV incorporates a non-uniform occupancy map, thereby simulating an energy distribution for states in data-type III. Detailed information pertaining to the construction of each of these three data types is provided in the Supplementary Material Section C.

## III. Analysis of Embeddings

In the following, the most significant findings of our heuristic analysis of the embeddings of numerous PD manifolds [41]—performed over three state spaces (SS_1_, SS_2_ and SS_3_) and four data-types, using both linear and nonlinear dimensionality-reduction methods—are presented. In sum, these discoveries provide a solid rationale for the strategies devised in the ESPER method described below.

As a note on the choice of dimensionality-reduction method, overall, the results of our analysis using PCA and DM were virtually identical, unless otherwise stated. Here we describe the embeddings achieved via DM, as is standard in the founding ManifoldEM methodology. A summary of both the DM and PCA approach is provided in Supplementary Material Section D, where we define parameters such as the Gaussian bandwidth (*ε*) used in the Gaussian kernel (12), and introduce the previously-established *double-filtering* kernel [1].

### Analysis of Data-type I.

**Fig. 1.**
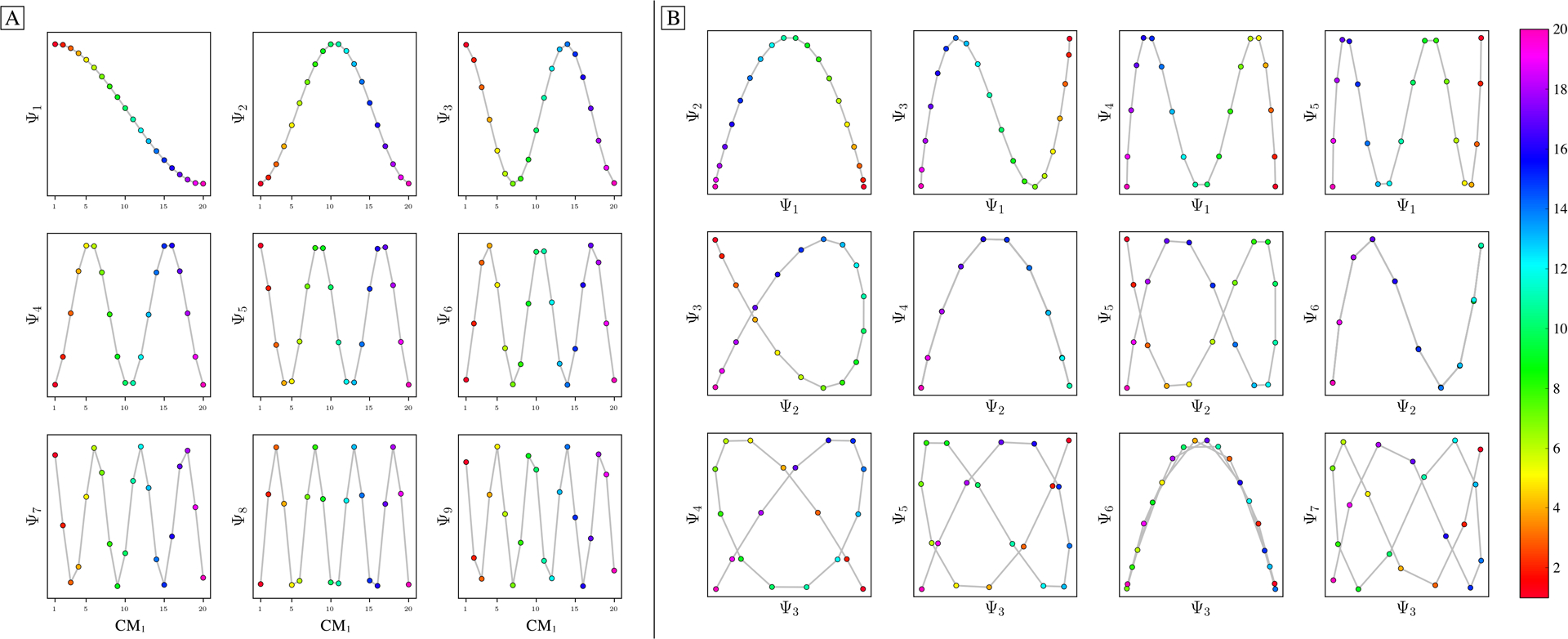
Analysis of eigenfunctions for PD_1_ in SS_1_ from data-type I. On the left (a) are the sinusoidal forms *ψ_k_* that emerge when points (corresponding to images) in each eigenvector are ordered precisely in the sequence in which the ground-truth CMs were constructed. Regardless of any knowledge of such a sequence, the composites of these eigenvectors will always form well-defined geometries (via the Lissajous curves), as shown in (b). In the first row are the Chebyshev polynomials of the first kind, of which the parabola {Ψ_1_ × Ψ_2_} is the simplest mapping of the conformational information present.

We first generated a different embedding for each of several PD manifolds in SS_1_, with each of the resultant point clouds containing a collection of points corresponding to images depicting conformational states from CM_1_. A distinct pattern emerged (Fig. 1(b)) when examining the embedding in terms of its set of 2D eigenvector subspaces {Ψ_*i*_ × Ψ_*j*_} where *i* < *j*, which revealed conformational signal following the Lissajous curves [46]

(1) $$L_{p,q}=\left\{\text{cos}(p\pi x)\times \text{cos}(q\pi x)|x\in [0,1];p<q\in {\mathbb{Z}}^{+}\right\}.$$

In (1), *x* is the conformational coordinate represented by a number in the interval [0, 1]. The appearance of these *L*_*p,q*_ curves—which are the Cartesian products (symbolized by ×) of two sinusoids—aligns with the known attributes of the LBO approximated by DM. Specifically, the functions

(2) $$\psi_{k}=\left\{\text{cos}(k\pi x\}|x\in [0,1];k\in {\mathbb{Z}}^{+}\right\}$$

are the canonical eigenfunctions of the LBO on the interval [0, 1] subject to Neumann boundary conditions [47]. We were able to directly observe these individual cosines in each PD embedding by ordering the indices of points in each eigenvector along the ground-truth CM_1_ sequence of states (Fig. 1(a)). When this procedure was repeated for SS_2_ and SS_3_—independently for each CM present—we observed that for *n* degrees of freedom in a given data set, there are *n* independent sets of sinusoids *ψ*_*k*_. Each of these sets $\left\{\psi_{k}^{\gamma }|\gamma \in {\mathbb{Z}}^{+}\leq n\right\}$, denoted by an index *γ* per degree of freedom, are interspersed throughout the leading eigenvectors.

**Fig. 2.**
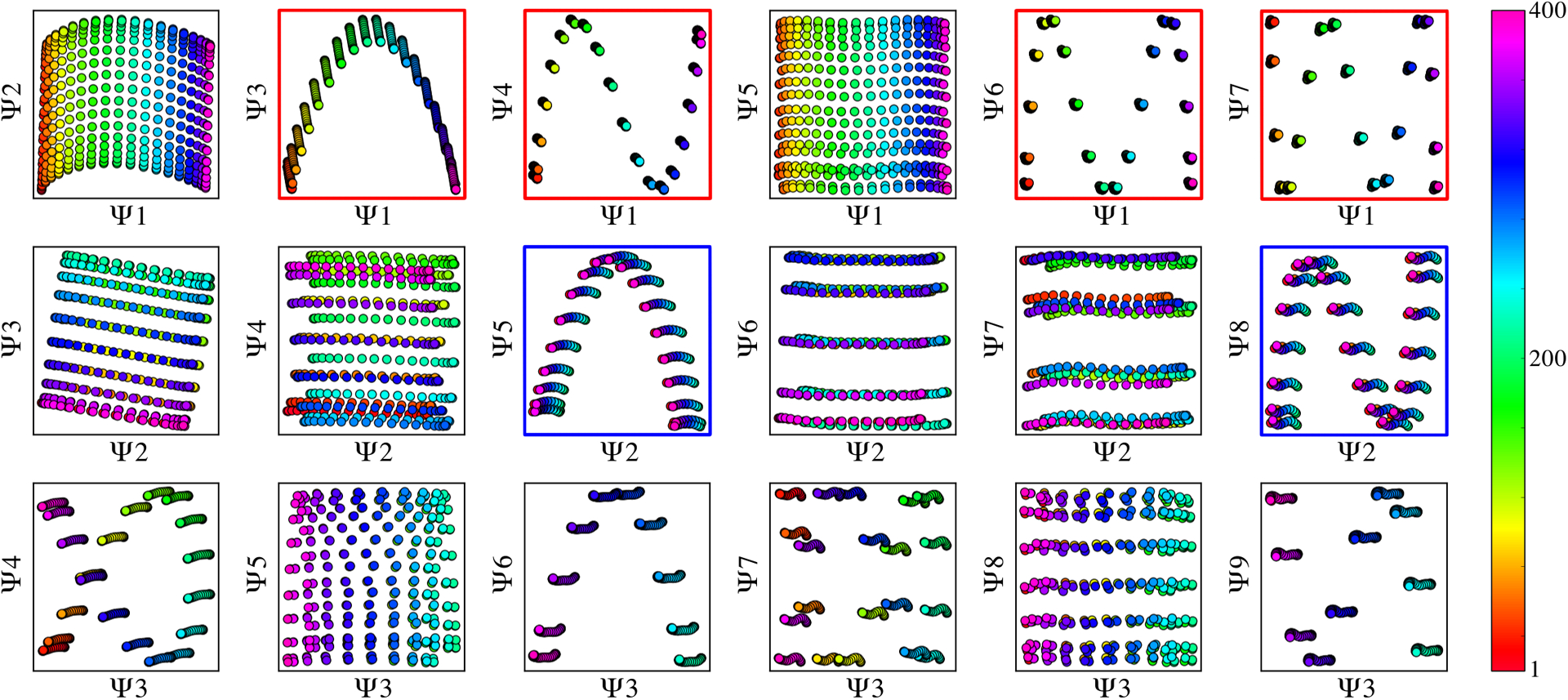
The spectral geometry within a subset of 2D subspaces for PD_1_ in SS_2_ is shown, as generated via DM. As seen for *n* > 1, (1) is significantly more complex, with hypersurfaces intermixed. The color map is defined to match the indices of points spanning CM_1_, such that CM_2_ points are approximately uniform in color map value (multiples of 20, overlaid).

While we can view each $\psi_{k}^{\gamma }$ individually, given privileged knowledge of the ground-truth sequence of states [41], this does not apply for experimental data, since points arrive in a random sequence and will also include missing or duplicate states. However, since the points in each $\psi_{k}^{\gamma }$ are always scrambled in the same way in all eigenvectors, we can instead rely on the composite of any two eigenvectors to always manifest a readily identifiable form. For these composites, we found that CM information is portrayed most simply (without overlap) along a specific subset of the *L*_*p,q*_ curves, and that for each CM, only a single 2D subspace was required to recapitulate ground truth. The eigenvectors {Ψ_*i*_ × Ψ_*j*_} of this essential subspace are defined by the Cartesian product of the first two eigenfunctions {*ψ*_1_ × *ψ*_2_}of the respective CM, forming a parabola (Fig. 2). In this projected view, states differing in coordinates that are orthogonal to the projection plane—and thus describe ulterior CM information embedded on a higher-dimension surface—overlap. We note that this finding stands in contrast to the previous ManifoldEM methodology, which starts with a single eigenvector Ψ_*i*_ from the initial embedding for mapping a given CM [1]. Later in our analysis, we will demonstrate the difference and its consequences.

As can be seen in Fig. 2, the parabola-housing 2D subspaces corresponding to CM_1_ and CM_2_ are {Ψ_1_ × Ψ_3_} and {Ψ_2_ × Ψ_5_}, corresponding to $\left\{\psi_{1}^{1}\times \psi_{2}^{1}\right\}$ and $\left\{\psi_{1}^{2}\times \psi_{2}^{2}\right\}$, respectively. These parabolas are a minority intermixed among a majority of 2D subspaces displaying the image sequence in a variety of more complicated spatial patterns. For example, {Ψ_1_ × Ψ_2_}displays both CM_1_ and CM_2_ content on a top-down projection of a parabolic surface—corresponding to $\left\{\psi_{1}^{1}\times \psi_{1}^{2}\right\}$—whereas {Ψ_3_ × Ψ_4_} charts CM_1_ information along an alpha-shaped trajectory $\left\{\psi_{2}^{1}\times \psi_{3}^{1}\right\}$. Great care must be taken to identify the highly-informative CM parabolas present, while avoiding subspaces where CM information is obfuscated.

The ability to do so is worsened by the additional presence of 2D subspaces displaying higher-order *L*_*p,q*_ parabolas (such as {Ψ_3_ × Ψ_6_} corresponding to $\left\{\psi_{2}^{1}\times \psi_{4}^{1}\right\}$) which deceptively repeat a conformational motion one or more times (i.e., multivalued) within one span of the parabolic trajectory. We denote these higher-order parabolas as *harmonics*, which do not preserve topological structure (i.e., non-injective surjections [48]) and must be avoided when mapping a CM. This is a problem that becomes more challenging for data sets with multiple degrees of freedom, which was not addressed in an automated way in the founding ManifoldEM methodology.

We next describe the major differences observed between the distributions of point clouds corresponding to different PDs. Naturally, as each 2D projection of the molecular machine provides an incomplete representation of the underlying 3D density map, depending on the type of motion as viewed in the PD under investigation, ground truth is preserved to different degrees. The effect of this *PD disparity* was present in all embeddings we analyzed, and especially those from data sets simulated with more than one degree of freedom. The most dominant characteristic was an apparent rotation of the point clouds in each subspace, as seen subtly in the 2D subspaces shown in Fig. 2 (e.g., {Ψ_2_ × Ψ_5_}). In other PDs, the effect can be more drastic, with each projected CM parabola appearing more like the projection of a rotated parabolic surface.

Through an analysis of how the canonical eigenfunctions on a rectangular domain transform as the data type is translated step-wise from atomic models to 3D density maps to 2D projections [41], we found that the cause of these rotations was tied to the projection of 3D macromolecular content in a given PD. In close approximation, a given eigenvector Ψ_*i*_ is a linear combination of *n* canonical eigenfunctions $\left\{\text{cos}\left(k\pi x_{\gamma }\right)|k\in {\mathbb{Z}}^{+}\right\}$, each corresponding to a degree of freedom $x_{\gamma }\subset {\mathbb{R}}^{n}$. As an example from our analysis using SS_2_, we show that the leading Ω_PD_ eigenfunctions appear in the form

(3) $$\Psi_{i}=\text{cos}(\theta )\text{cos}(v\pi x)+\text{sin}(\theta )\text{cos}(w\pi y)=A\psi_{v}+B\psi_{w}$$

Using this explicit expression, we are able to near-perfectly approximate the heuristically-derived embeddings [41]. Further, the sum of the squared coefficients is conserved across pairs of eigenvectors, such that the base functions $\Psi_{i}^{\prime }=\psi_{v}$ and $\Psi_{j}^{\prime }=\psi_{w}$ can be expressed as a rotation Ψ = ***R***^*T*^ Ψ′, with form

(4) $$\left[\begin{array}{c} \Psi_{i}(\theta )\\ \Psi_{j}(\theta ) \end{array}\right]=\left[\begin{array}{c} \text{cos}(\theta )\psi_{v}+\text{sin}(\theta )\psi_{w}\\ -\text{sin}(\theta )\psi_{v}+\text{cos}(\theta )\psi_{w} \end{array}\right]$$

From our analytical expression, it is clear that, depending on the PD, CM information—pertaining to each of the system’s degrees of freedom—will lie on some linear combination of the embedded manifold’s orthogonal eigenvectors. We denote this feature as a result of *eigenfunction misalignments,* which are neither described nor accounted for in the original ManifoldEM framework, and explain some of its previously-documented problems [35], [36], [38].

### Analysis of Data-type II.

**Fig. 3.**
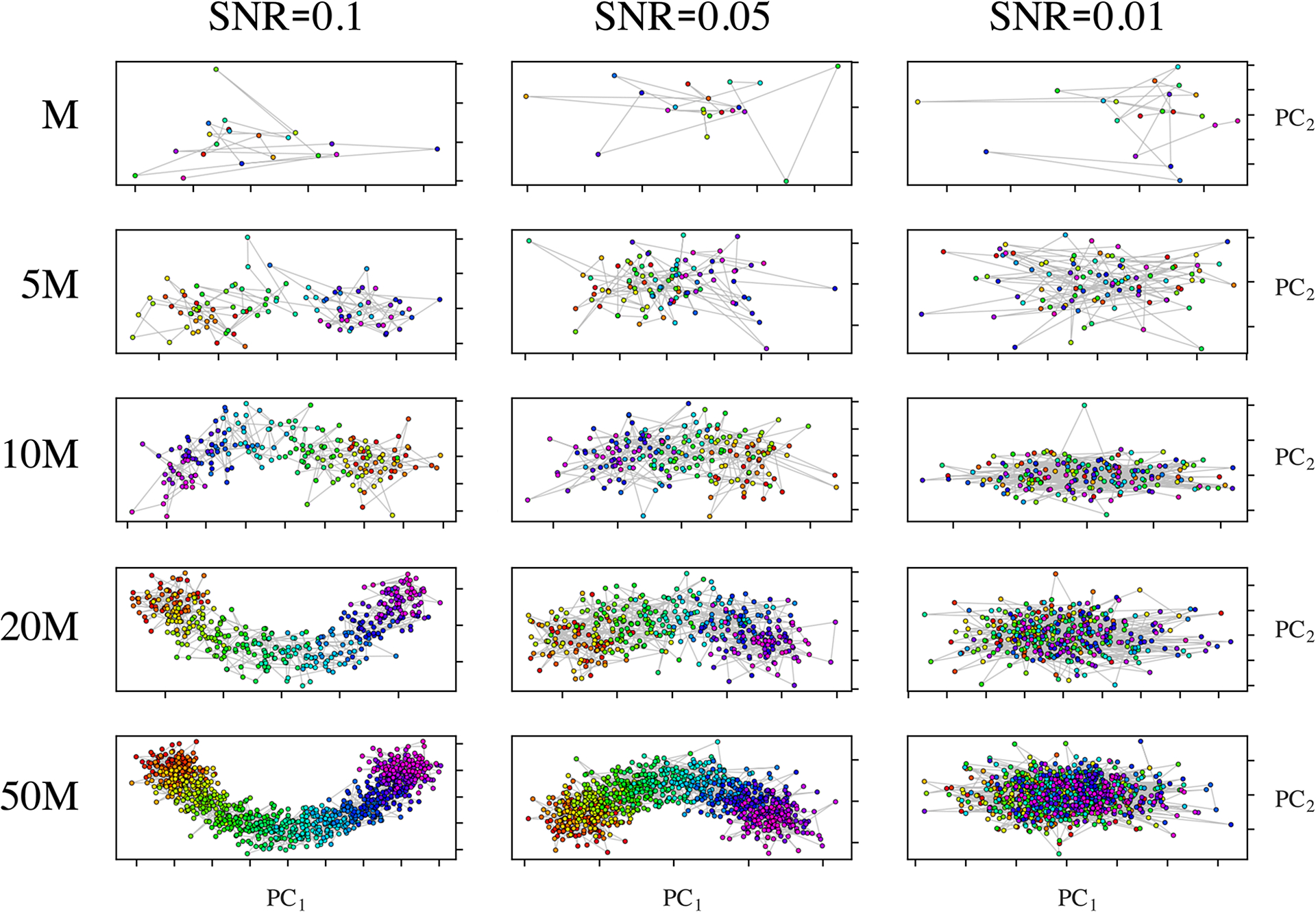
Set of {PC_1_, PC_2_} subspaces produced by PCA from PD_1_ images in SS_1_ over a range of SNR values and levels of state space coverage.

As finite SNR is an important attribute of any experimental data set, we next sought to understand how the structure of the PD embeddings change with varying SNR (Fig. 15) and state space coverage. For both PCA and DM as dimensionality-reduction technique, the fidelity of the resulting spectral geometry to the state space ordering decayed with increasing noise level. Overall, the behavior of the embeddings from each PCA and DM became increasingly similar as the SNR was decreased (Fig. 3).

At the same time, we investigated the effects of varying state space coverage across several SNR regimes, and its effects on the robustness of the corresponding embeddings. For this study, we used the 20 images in PD_1_ representing SS_1_ (i.e., one full range of conformational motion), and varied both the number of times (*τ*) these *M* = 20 ground-truth states were duplicated as a group—with each instance having a different realization of additive Gaussian noise—and the SNR of each image therein. Here, Gaussian noise of constant variance was applied for each SNR regime and uniquely added to each of the *τM = N* images independently. An excerpt from the results of our analysis is shown in Fig. 3, where a highly structured pattern emerged. Specifically, when noise at increasing levels was added to each image (decreasing SNR), increasingly larger values of state occupancy were required to reestablish a coherent structure in the spectral geometry. We found that as the value of *τ* is increased, there exists a lower threshold (τ_*c*_) such that the arrangement of points in the embedding is in highest achievable consistency with its ground-truth state space. In other words, there is a fixed amount of coverage that is sufficient.

**Fig. 4.**
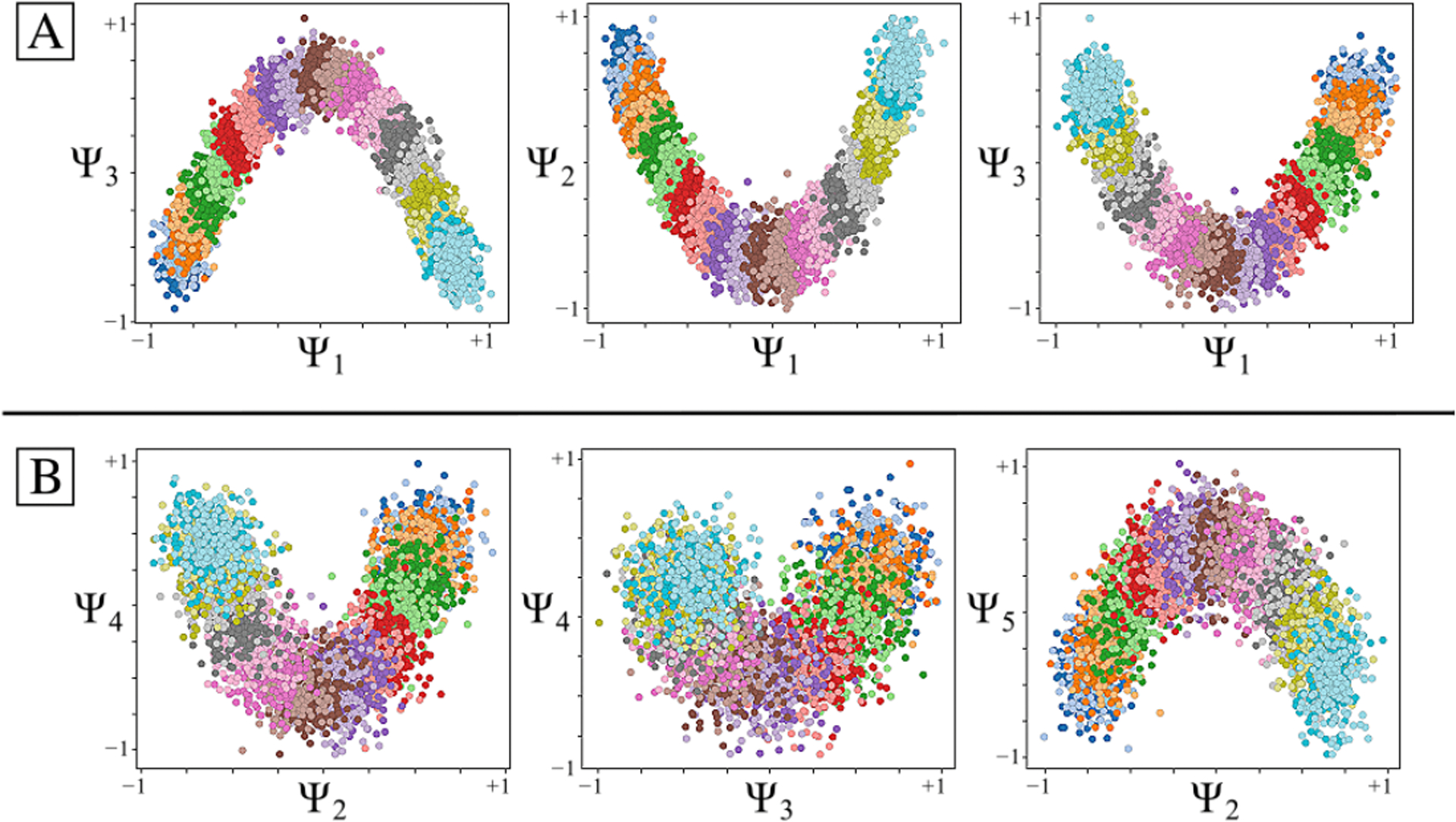
Comparison of CM subspaces for three PDs generated from data-type II. Here, SNR of 0.1 and *τ* = 10 is used, with embeddings achieved via PCA (similar trends were also found for DM). The coordinates within each point cloud are colored to indicate their ground-truth CM state assignment, such that each point belongs to one of the 20 CM bins, and each bin contains 200 points (with the same coloring scheme used regardless of CM). CM_1_ and CM_2_ subspaces for three randomly-oriented PDs are shown in (a) and (b), respectively, so as to emphasize the variability in features prevalent in embeddings obtained from noisy images.

This trend is demonstrated in Fig. 4 for three PDs where CM_1_ is highly pronounced (with arm motion along the projection plane), while CM_2_ is visually obscured to different extents. Due to these relations, the point clouds corresponding to CM_2_ appear far less structured than their CM_1_ counterparts. In general, due to PD disparity, we found that the characteristics of each CM-parabola can be seen to vary significantly depending on viewing direction. The variations include average width, length, density, trajectory, and spread of data points in each parabolic point cloud, with aberrations occurring most frequently in CM subspaces generated from PDs where the apparent range of the given CM is diminished. As a result, while the CM subspaces for all PD manifolds carry reliable content for recovery of 3D density maps along a conformational trajectory, certain clusters of PDs ⊂ *S*^2^ offer less reliable geometric structure for accurately estimating occupancies of CM states therein.

### Analysis of Data-type III.

We finally analyzed the PD manifolds obtained from image ensembles generated with experimentally-relevant CTFs and SNR, as detailed in Supplementary Section C. Specifically, we tested the performance of the CTF double-filtering kernel [1], and found a noticeable inward-curling at the ends of the resulting CM subspace parabolas. Notwithstanding this artifact, the double-filtering kernel was successful in preserving the most important aspects of the manifold, and proved superior to alternative techniques explored, such as embedding using the standard kernel from sets of CTF-corrected images.

### Additional Considerations.

In the following section, several considerations are provided pertaining to the relevancy and breadth of this heuristic analysis. Our preceding analysis is focused on data models originating from molecules undergoing collective rigid-body motions, which we believe are sufficient for most molecular machines, but may fall short of addressing instances involving more complex situations. This is especially the case for those machines entailing the concerted binding and release of ligands, which naturally require a separate state space for each possible combination of the machine with its binding partners. For such a situation, a similar heuristic analysis could be conducted using synthetic models occupying two or more state spaces.

**Fig. 5.**
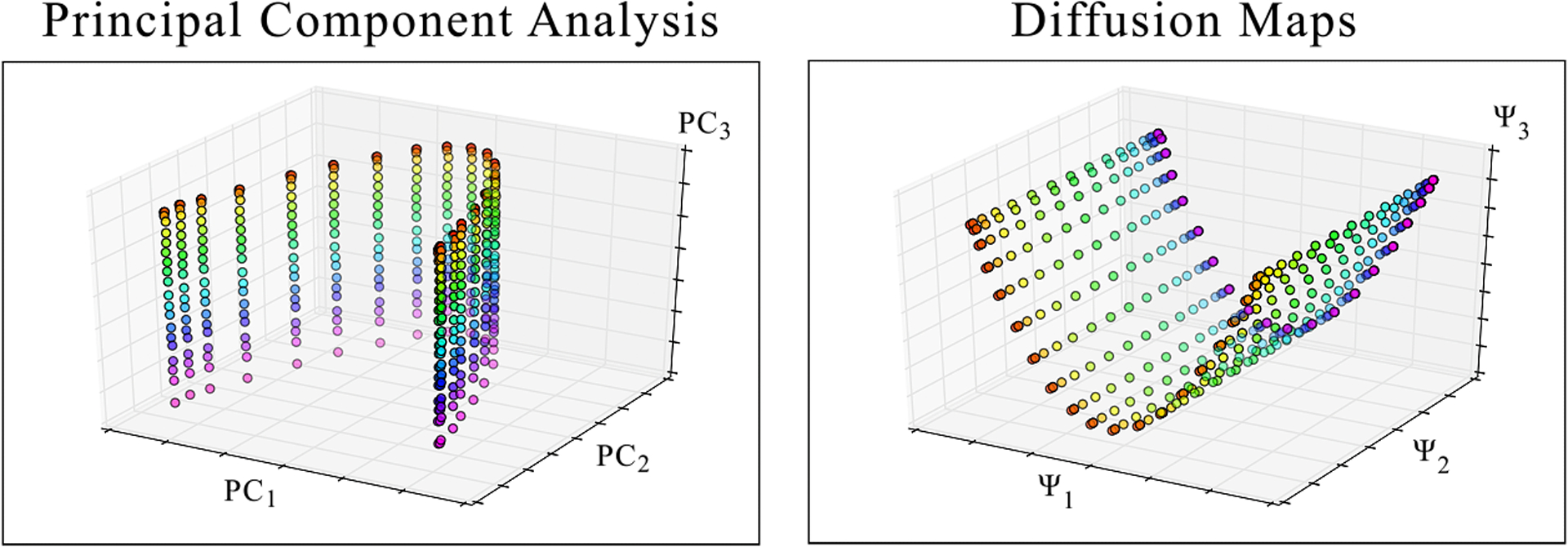
Comparison of PCA and DM embeddings of the 400 images of the “mouth-wing” toy model in SS_2_ from a given PD. The anticipated 20 × 20 parabolic surface is obtained by both techniques. Of note, the points in the PCA embedding have a slightly-less uniform distribution than those in the DM embedding, suggesting that DM better approximates intrinsic relationships in the data. Overall, these results closely match those obtained from application of PCA and DM on the Hsp90 SS_2_ synthetic continuum.

For completeness, we further tested the ability of PCA and DM to correctly embed PD manifolds formed from models exercising more complex motions. For this purpose, an ensemble of projections of the mouth-wings toy model (Fig. 14) was generated as described in Supplementary Material Section B. Compared to the synthetic framework used to generate the Hsp90 data set, this workflow provides a radically different approach, and incorporates concerted translation of atoms along different directions and magnitudes in the mouth section, which differ from domain rotations. Nonetheless, the embedding of these mouth-wings images still manifested all essential geometric characteristics previously detailed for Hsp90: presenting SS_2_ across a parabolic sheet (Fig. 5), as expected. Although the procurement of the mouth-wings model is nowhere near an exhaustive coverage of possible motion modalities, we believe the correspondence between its outputs and those of the independently-designed Hsp90 data set establishes some generality for our discoveries.

Finally for consideration, we have only dealt here with synthetic models that specifically exhibit each of their domain motions along an independent and mutually unrestricted sequence of quasi-continuous states. All *n*-wise combinations of these bounded intervals (one for each CM) produce an *n*-dimensional shape with a rectangular boundary. As a prerequisite to our conditions for adequate continuum reconstruction, the minimum coverage of cryo-EM images must be obtained (i.e., as achieved near τ_*c*_) so as to effectively fill in this hypercube. For experimental conditions where each state occurs with a given frequency as dictated by its underlying free energy, this condition must be met for the least abundant states in the data set. As it turns out, this condition must only be met for a handful of PDs at minimum, to be described at length in our discussion.

Once this condition is met, we have further shown that the corresponding Laplacian eigenfunctions are well defined for the hypercube domain [41]. However, in general, analytically solving the Laplacian for any arbitrary boundary is impossible. Eigenfunctions can change drastically depending on the boundary, and are analytically only known for certain elementary shapes, such as rectangles, discs, ellipses and special triangles [47]. On the other hand, geometric machine learning approaches can obtain solutions numerically, in principle for any boundary. However, such geometric machine learning methods still require the boundary to be known *a priori*. For systems with unknown boundaries, the problem is intractable.

As the set of all possible molecular machines is unfathomably complex, it is unlikely that one single algorithm could ever be so versatile as to anticipate every possible instance. Instead, we are interested in casting a wide enough net so as to capture the dynamics of a large portion of these systems, which we surmise operate within rectangular boundaries of an *n*-dimensional latent space of relatively-rigid multi-body motions. However, one can still imagine all sorts of other situations, such as a system where one domain blocks—via *steric hindrance*—another domain from its full range of motion in a specific region of the state space. We will return to this topic after the introduction of the ESPER method in the following chapter.

## IV. The ESPER Method

Having conducted our detailed heuristic analysis, we now describe the ESPER method for recovery of conformational continuum from each Ω_PD_ embedding. Our method has been designed to leverage the geometric features discovered upon applying ManifoldEM to synthetic data and address some of the problems encountered before; later we will detail caveats for data obtained from experiment. ESPER includes several novel strategies required to form the final free-energy landscape and corresponding 3D movies. These strategies include realignment of eigenfunctions, partitioning of 2D subspaces, and compilation of CM information on *S*^2^. In the following, each of these strategies will be outlined in turn.

### Eigenfunction Realignment.

Previously, we described how the observed CM eigenfunctions may be misaligned with respect to the ideal eigenfunctions of the LBO, such that the correct sequence of conformational information is obfuscated along the given eigenvectors, to different degrees depending on PD. Since these misalignments are due to the change in apparent PD-dependent interatomic distances, they are inevitable and pose a fundamental problem that must be addressed. As a remedy, the ESPER method aims to isolate the set of orthogonal sinusoids representing each CM in their complete form within each Ω_PD_ eigenbasis.

In our previous exposition [41], we show that by use of appropriate rotation operators *R*_*i,j*_, the canonical eigenbasis for each CM can be recovered. As a result of this decoupling of eigenfunctions onto a set of appropriate eigenvectors, each corresponding parabolic surface becomes aligned within its 2D subspace, and the projected structure is again that of a single parabola carrying information about a single CM along its curve. Thus, as long as each parabolic trajectory corresponding to a given CM is aligned with the plane of an independent 2D subspace, we can restrict our study to an analysis of only a few essential subspaces; one for each degree of freedom.

**Fig. 6.**
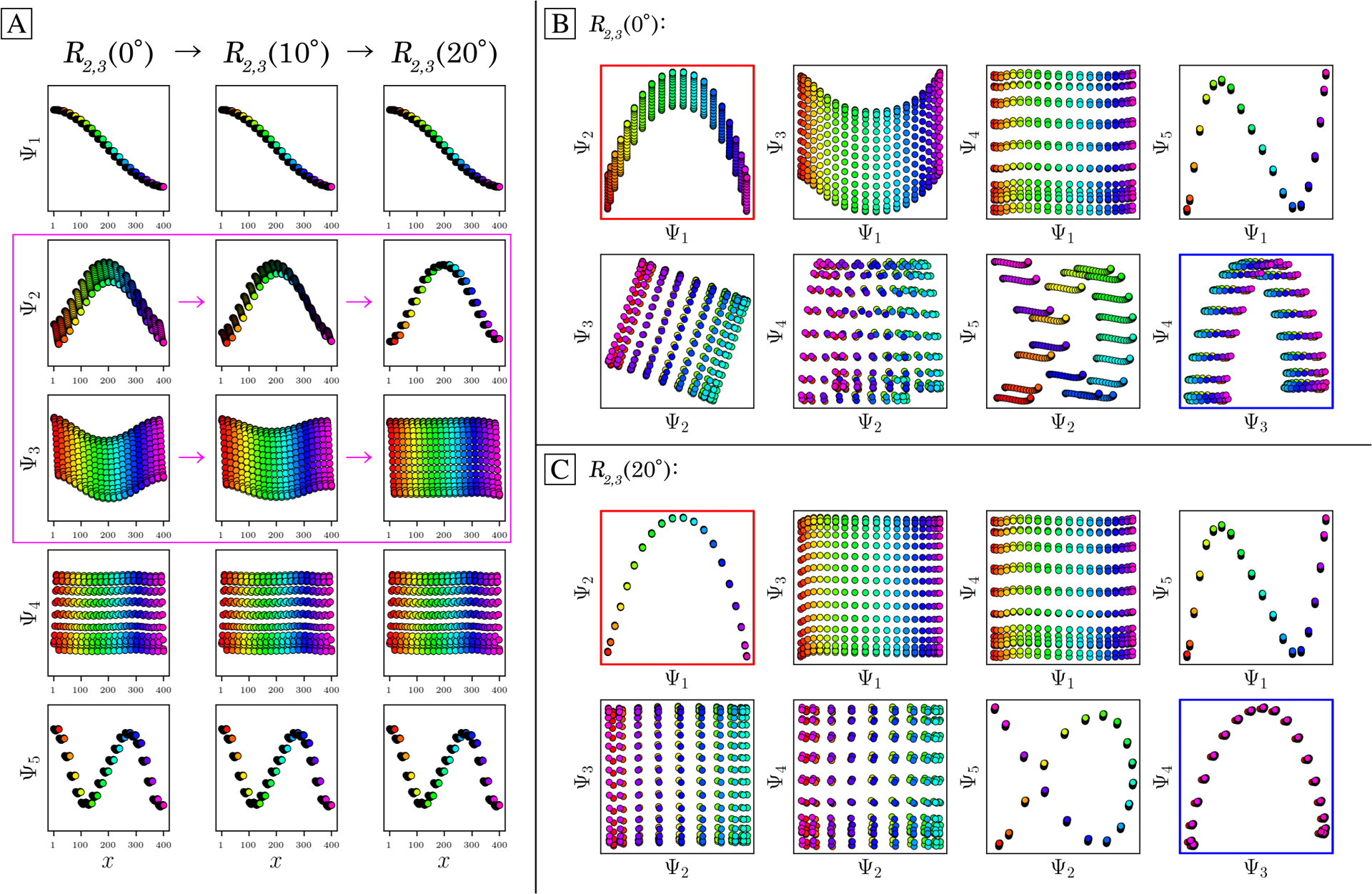
Application of a 5D rotation matrix *R*_2,3_(*θ*) on an initially misaligned Ω_PD_ embedding generated from SS_2_ in data-type I. The three columns in (a) display the individual eigenfunctions (as plotted by indices corresponding to the CM_1_ frame of reference) before the *R*_2,3_(*θ*) rotation is applied, at *R*_2,3_(10°), and finally at *R*_2,3_(20°), respectively. Note that *R*_2,3_(20°) maximally decomposes Ψ_2_ and Ψ_3_ into unique sinusoids (recalling that the planar distribution in Ψ_3_ is in fact a sinusoid when visualized in the CM_2_ frame of reference, and vice versa for Ψ_2_). The before and after effects of these rotations on the Lissajous curves can likewise be seen in (b) and (c), respectively. Applying *R*_2,3_(20°) properly orients both parabolic surfaces corresponding to CM_1_ and CM_2_ (denoted with red and blue boxes, respectively), such that the eigenvectors are orthogonally aligned with the eigenbasis of the CMs.

As a demonstration of this technique—termed *eigenfunction realignment*—Fig. 6(a) shows the eigenvectors (reordered along CM_1_) for a highly-misaligned PD eigenbasis from SS_2_ in data-type I. As seen in the first column, while $\Psi_{1}=\psi_{1}^{1}$, $\Psi_{4}=\psi_{2}^{2}$ and $\Psi_{5}=\psi_{3}^{1}$ are in agreement with expectations, $\Psi_{2}=\psi_{2}^{1}$ and $\Psi_{3}=\psi_{1}^{2}$ appear heavily deformed. (Recall that the planar distributions are in fact sinusoids when visualized in the CM_2_ frame of reference). As a direct consequence, any subspace composed in combination with Ψ_2_ or Ψ_3_ will be misaligned with respect to its ideal form (Fig. 6(b)).

ESPER is designed to correct for these misalignments using orthogonal transformations. Specifically, we apply a rotation operator represented by a *d* × *d* matrix *O* of sufficiently large dimensions, as required for encompassing all CM subspaces, to single-handedly reorient all aberrant surfaces in their respective 2D subspaces. The matrix *O* can be represented by the product of *d(d* − l)/2 rotation sub-matrices *R*_*i,j*_, with each sub-matrix parameterized by a unique angle and operating on a specific plane. The results of this operation in a selected example can be seen in Fig. 6(b) and (c); before and after applying a 5D rotation matrix, respectively.

For the specific case of the 5D rotation matrix, there exist 10 rotation sub-matrices in total, with each corresponding to a specific rotation on the eigenbasis. Of these 10 matrices, we found that only one had to be altered for this case to achieve the results shown, having general form

(5) $$R_{2,3}(\theta )=\left[\begin{array}{ccccc} 1 & 0 & 0 & 0 & \cdots \\ 0 & \text{cos}(\theta ) & -\text{sin}(\theta ) & 0 & \cdots \\ 0 & \text{sin}(\theta ) & \text{cos}(\theta ) & 0 & \cdots \\ 0 & 0 & 0 & 1 & \cdots \\ \vdots & \vdots & \vdots & \vdots & \ddots \end{array}\right]$$

As this *R*_**2**,**3**_*(θ)* operator corresponds to transformations performed solely on Ψ_2_ and Ψ_3_ (row 2 and 3 of the rotation matrix, respectively), eigenvectors previously identified as problematic are thus isolated. The result of this transformation on the full set of eigenvectors can be seen in the three columns of Fig. 6(a), which visualize the *R*_**2**,**3**_*(θ)* rotation under 0°, 10° and 20°, respectively. Only Ψ_2_ and Ψ_3_ undergo change, as expected. After this operation, the initially entangled sinusoidal information contained in part between Ψ_2_ and Ψ_3_ is maximally separated between both eigenvectors, ultimately resulting in the alignment of all corresponding surfaces with their 2D subspaces (Fig. 6(c)), as desired. We also show the effects of applying a 4D rotation on SS_2_ in data-type II in Movie 3, where only one of the six possible *R*_*i,j*_ was altered to realign both CM_1_ and CM_2_ parabolas to the plane of their respective 2D subspaces.


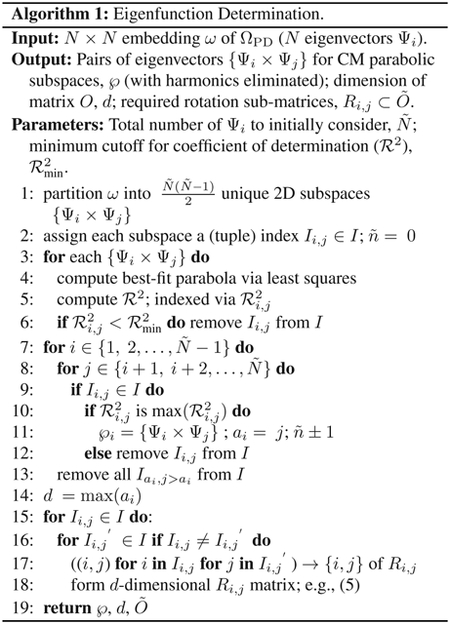


To generalize this solution for any Ω_PD_ embedding, there are thus three unknowns: (*i*) the dimensionality *d* of the matrix *O*; (*ii*) the required rotation sub-matrices *R*_*i,j*_; and, for each of these *R*_*i,j*_, (*iii*) the rotation angle *θ*. After careful observation of all PDs across numerous data sets, we have determined that the dimensionality *d* and rotation operators *R*_*i,j*_ required are linked to the indices of eigenvectors housing each CM parabola. As a consequence, we need to first determine these CM subspaces, which can be identified by a systematic comparison of least-squares fits, while eliminating subspaces housing parabolic harmonics. The pseudocode of the *eigenfunction determination* procedure is given in Algorithm 1.

As a result of Algorithm 1, eigenvectors housing CM subspaces ℘ are identified (line 1.11)—while excluding the possibility of parabolic harmonics (line 1.13)—with $\frac{\tilde{n}!2}{(\tilde{n}-2)!}$ essential *d*-dimensional *R*_*i,j*_ operators defined (line 1.18). The rationale for removal of harmonics can be easily understood, since any 2D subspace formed in part by an eigenfunction corresponding to a known CM parabola cannot combine to form some other orthogonal CM parabola. Once these CM subspaces are known, we approximate the third unknown—the rotation angle—using 2D histograms. In the case of noisy data, as each 2D subspace is rotated by a given *R*_*i,j*_ (*θ*), it exhibits a unique profile that can be characterized by a sequence of 2D histograms on that subspace, with one 2D histogram per each rotation angle *θ*. When we plot the number of nonzero bins in the corresponding 2D histogram as a function of *R*_*i,j*_ (*θ*), the minimum in this distribution corresponds to the angle required to properly counter-rotate each 2D subspace by the current operator (Movie 4). The pseudocode of the *eigenfunction realignment* procedure is provided in Algorithm 2.


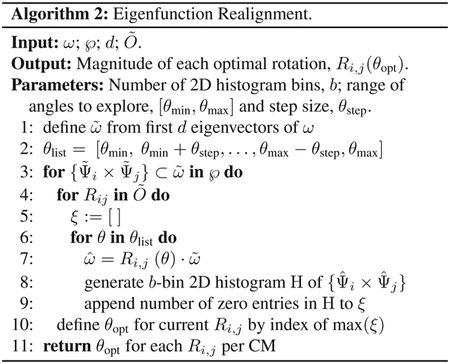


To good approximation, the *d*-dimensional rotations performed for each *R*_*i,j*_ operator in Algorithm 2 realign the essential eigenfunctions of each Ω_PD_ CM subspace. An example visualization of this entire workflow, demonstrating the performance of Algorithm 1 and Algorithm 2 applied on a SS_2_ embedding from data-type II, is provided in Movie 5. We additionally perform a final least-squares fit ${\hat{\Psi }}_{\text{fit }}$ on each rotated CM subspace. For data-type III, we found an implicit equation of a general conic section to be most flexible, defined by a polynomial of degree two

(6) $$ax^{2}+bxy+cy^{2}+dx+ey+f=0,$$

which allows for the possibility of parabolic-like trajectories with elliptic or hyperbolic features. This flexibility is essential for fitting parabolic-like point clouds with inward curling near the boundaries, as was observed for manifolds formed by images modified by the CTF.

### Subspace Partitioning.

**Fig. 7.**
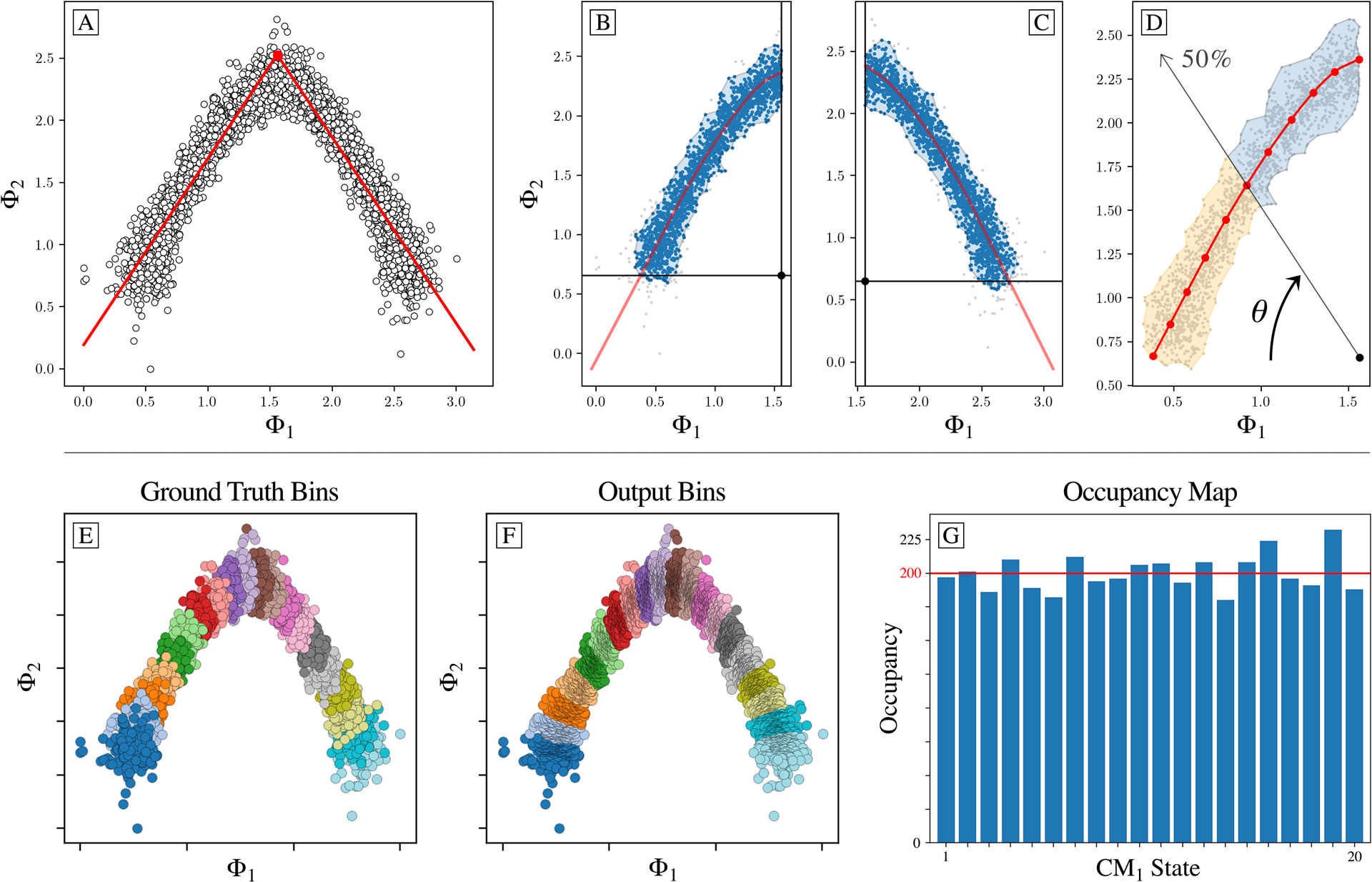
Overview of our *subspace partitioning* procedure for extracting sequential conformational information from a given 2D subspace. For this example, a CM_1_ subspace from data-type II is shown. Subplots (a) through (g) display our algorithm’s outputs on the CM_1_ subspace of an arbitrary PD from data-type II. First, (a) shows the inverse-cosine transformation and corresponding preliminary fit using an absolute value function. Subplots (b) and (c) demonstrate the alpha-shape polygon and Φ_*fit*_ trajectory defined on each halved subspace, with an anchor point designated within the central alcove. In (d), a ray is shown passing from the anchor point through the point cloud. At the current angle *θ* shown, half of the area of the alpha shape has been traversed, demarcating the boundary between the 5^th^ and 6^th^ (of 10) CM_1_ bins. Subplots (e) and (f) compare the ground-truth bins—as visualized via the known sequence of images in each state—with the final output bins produced by the ESPER method. The 1D occupancy map is provided in (g), where the horizontal red line (200 images) represents the ground-truth occupancy assignment per CM_1_ state.

Once each CM subspace is identified and rotated for a single PD, we must next correct for the nonuniform rates of change along the parabolas, which arise innately as a result of taking the Cartesian product of sinusoids. As a remedy, we apply an inverse-cosine mapping on each CM eigenvector, which presents the coordinates of the respective eigenfunctions in a space with uniform rates of change, consistent with the ground-truth relationships between atomic-coordinate structures [41]. We will indicate any eigenvector Ψ_*i*_ under this transformation with the insignium Φ_*i*_. Each {Φ_*i*_ × Φ_*j*_} CM subspace is then partitioned into a set of contiguous equal-area bins, representing collectively a quasi-continuum of conformational states, as shown in Fig. 7.

The motivation for this approach stems from the analysis of PD disparity in the presence of noise, where it is observed that the ground-truth bins and overall area of each point cloud manifest in a variety of sizes depending on viewing angle. Our area-based point-cloud fitting approach is able to correctly chart spatial discrepancies while remaining unencumbered by changing densities (i.e., occupancies) along each trajectory. For partitioning of each CM subspace, we first use the alpha shapes algorithm [49] to define the overall area of each CM point cloud with a polygon—a generalization of the convex hull—representing the key features of its geometric shape (Fig. 7(b) and (c)). Next, using rays emanating from a point opposite the point cloud’s apex (Fig. 7(d)), we divide this polygon into a collection of contiguous sub-polygons of equal area (Fig. 7(f)). Each of these sub-polygons, in sequence, corresponds to one of the CM’s unique states, with the total number of points contained within each sub-polygon defining the corresponding state’s occupancy (Fig. 7(g)).

Since the points in any CM subspace that are aligned orthogonal to the respective projection plane describe ulterior CM information, averaging points together in that subspace only reveals the conformational information corresponding to the current CM. Hence, cryo-EM images assigned to each state can be averaged to generate each frame of the respective CM’s 2D movie. This process is then repeated for the 2D subspace where the second CM parabola resides, and so on for higher degrees of freedom. The pseudocode for the *subspace partitioning* procedure is given in Algorithm 3 in Supplementary Material Section G. The 2D movies obtained by this procedure for the example chosen can be found in Movie 6, showing both CM_1_ and CM_2_ captured along SS_2_ subspaces from images generated with SNR of 0.1 and τ = 5 via data-type II. A similar output can be found in Movie 7 for data-type IV.

### Conformation Compilation.

After aligning eigenfunctions and generating all 2D movies (one per CM for each PD), both the type of CM present in the 2D movie (e.g., CM_1_ or CM_2_) as well as its *sense* must be determined individually for each PD. This is a precondition for matching PD content globally on *S*^2^, since the ordering direction of states along a CM trajectory is arbitrary for each PD, due to arbitrary eigenfunction-polarity assignment inherent to any eigendecomposition [50]. While this information can be derived by visual assessment of 2D movies, a comprehensive automated strategy has also been developed using optical flow and belief propagation algorithms [37]. Once CM types and senses are assigned, the 2D movie of a given CM—housing indices of all images within its frames—can next be compiled together with all other 2D movies of that same CM across all PDs.

Following the ESPER method, we next generate an *n*-dimensional occupancy map by taking the intersection (overlap) of image indices corresponding to each combination of bins in the CM trajectories per PD. (Intuition for this procedure can be found in Supplementary Material Section G). Since the CM coordinates are intrinsically linked by the independent occurrence of image indices from the same PD image stack, this operation effectively reconstructs the *n*-dimensional hypersurface on which the images jointly reside. (If only one degree of freedom is desired, naturally no intersection is required). Next, image stacks—one for each state—are generated and paired with an alignment file that carries the input alignment and microscopy information for each image therein. These files can then be used as input for the 3D reconstruction (e.g., as can be performed by RELION [51]) of the molecule in each state in the compiled state space. A more detailed description of all preceding steps in the ESPER method is additionally available [41], including comprehensive Python code [52].

**Fig. 8.**
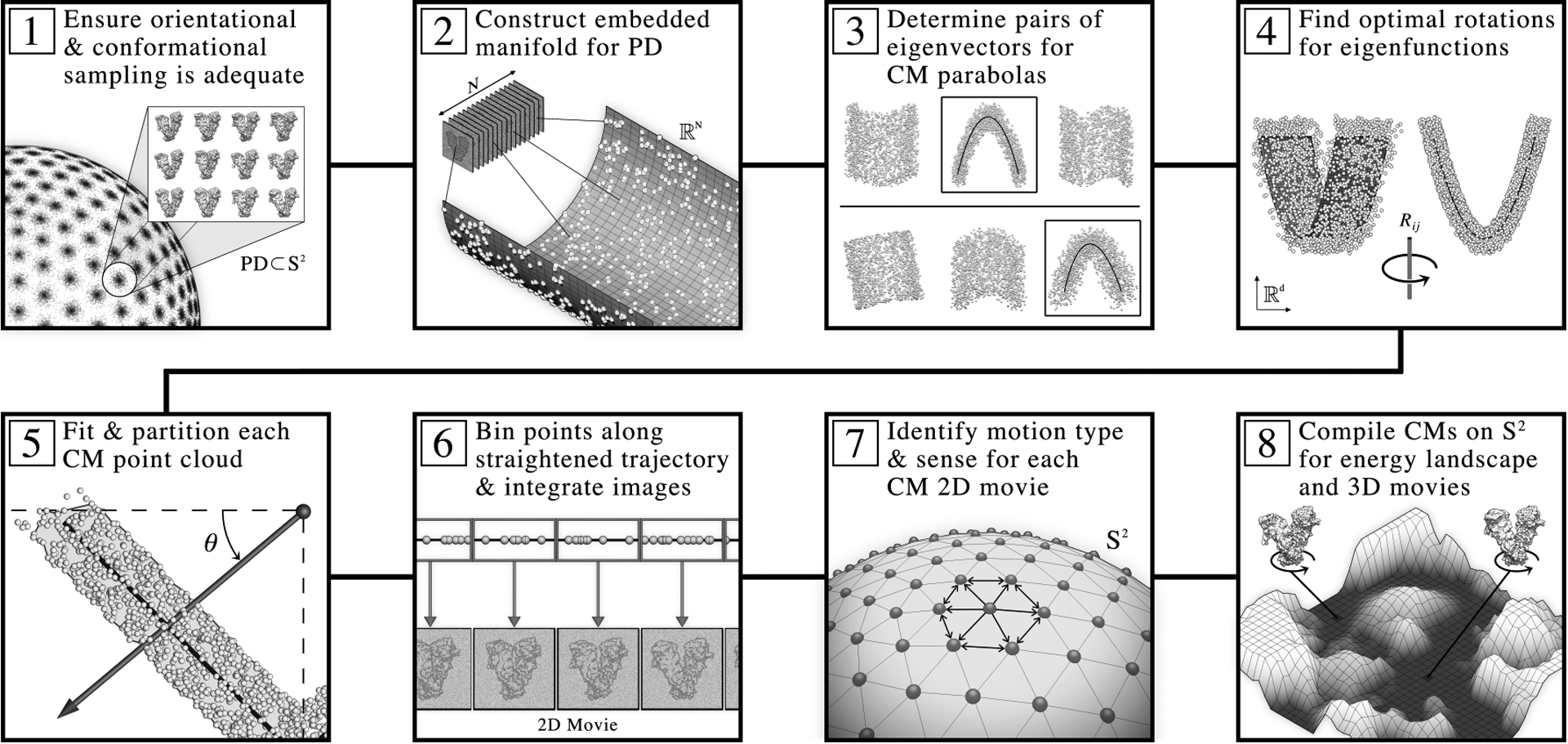
Schematic detailing the ESPER workflow for recovery of conformational continuum, as contained within the overarching ManifoldEM framework. For explanation of the individual steps, see main text.

Finally, to place ESPER within the context of the overarching ManifoldEM framework [1], we have provided a schematic in Fig. 8. Here, the ESPER method branches off from the ManifoldEM workflow after completion of step 2. While ManifoldEM next performs a series of steps required by NLSA (see Supplementary Material Section E for a brief summary), ESPER instead performs *eigenfunction realignment* (steps 3 and 4) and *subspace partitioning* (steps 5 and 6). The two methods meet again at step 7 to achieve reconciliation of PD manifolds across *S*^2^, before splitting off once again to form final outputs independently in step 8.

For our analysis of synthetic continua in each data-type, we note that the stipulation in step 1 of the workflow in Fig. 8 is satisfied, with all PD manifolds formed with sufficient qualities for the observance of parabolic point clouds. The performance of the ESPER method hinges on the presence of this geometric information, and, as we will next show with a direct comparison to NLSA, a great number of benefits emerge when it is available. After this comparison, we will more concisely quantify the conditions for parabolic point clouds during our analysis of experimental data. Additionally, in the event that only a subset of Ω_PD_ embeddings exist meeting these conditions, we will provide a strategy for alternating between use of ESPER and NLSA within the ManifoldEM framework.

## V. Results With Synthetic Data

**Fig. 9.**
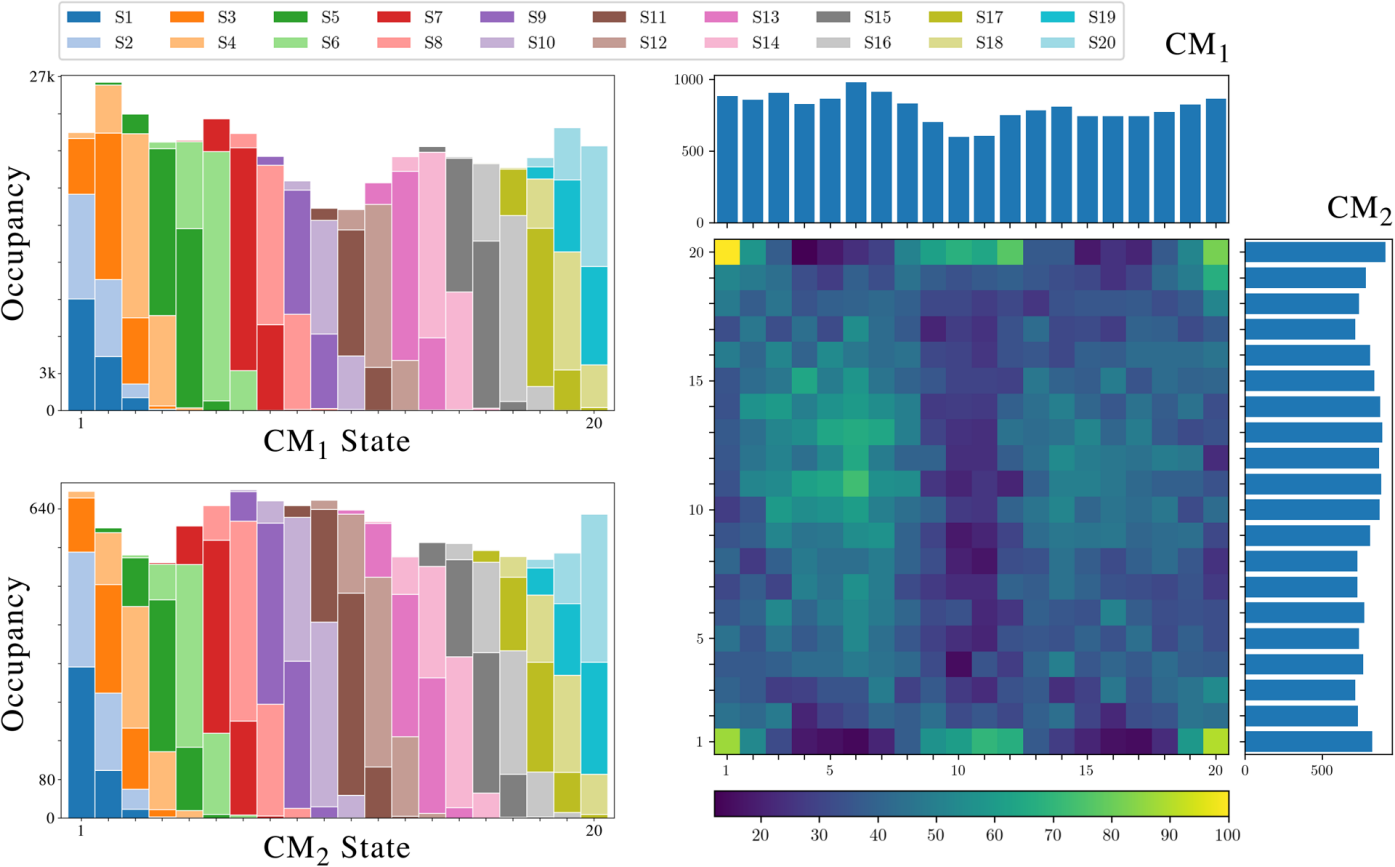
On the left, final occupancy maps for the 20 states in CM_1_ (top) and CM_2_ (bottom) are shown. The total number of images assigned to each state by use of the ESPER method is shown by the height of the corresponding bar, and the different colors represent how many of those assignments belong to which ground-truth states (as seen in the color keys above the figure), allowing an assessment of the true positive rate. On the right, the final 2D occupancy map for the 400 states formed by CM_1_ and CM_2_ is shown.

The results of applying the entire ESPER method on the 126 PDs from SS_2_ in data-type IV (with experimentally-relevant SNR and CTF) are shown in Fig. 9 and Movie 8. The former demonstrates the accuracy of occupancy assignments for comparison with Fig. 16 in Supplementary Material Section C, with the 2D occupancy map obtained via the intersection of image indices in all pairwise combinations of CM_1_ and CM_2_ bins (corrected for sense) in each of the 126 PDs. Overall, the results prove to be very accurate, with only subtle differences in occupancies near the boundaries of each CM, which manifest on the four corners of the 2D occupancy map. These discrepancies are mainly due to a combination of PD disparity, CTF-induced inward curling, and the vanishing derivatives of the DM eigenfunctions at the boundaries [22], [50], arising in each Ω_PD_ embedding. To circumvent issues stemming from inclusion of CM subspaces with poor geometric structure arising from PD disparity, we note that while all images are used for subsequent 3D reconstructions, only those occupancy assignments for CM subspaces above an ℛ^2^ threshold value (0.7) were integrated during this analysis. In Movie 8, the occupancy map without ℛ^2^ thresholding is shown, along with the corresponding final 3D density maps for an example trajectory (3D movie) from the compiled 2D state space. As can be seen, the ESPER outputs uphold the spatial relationships in the ground-truth CMs with striking accuracy.

**Fig. 10.**
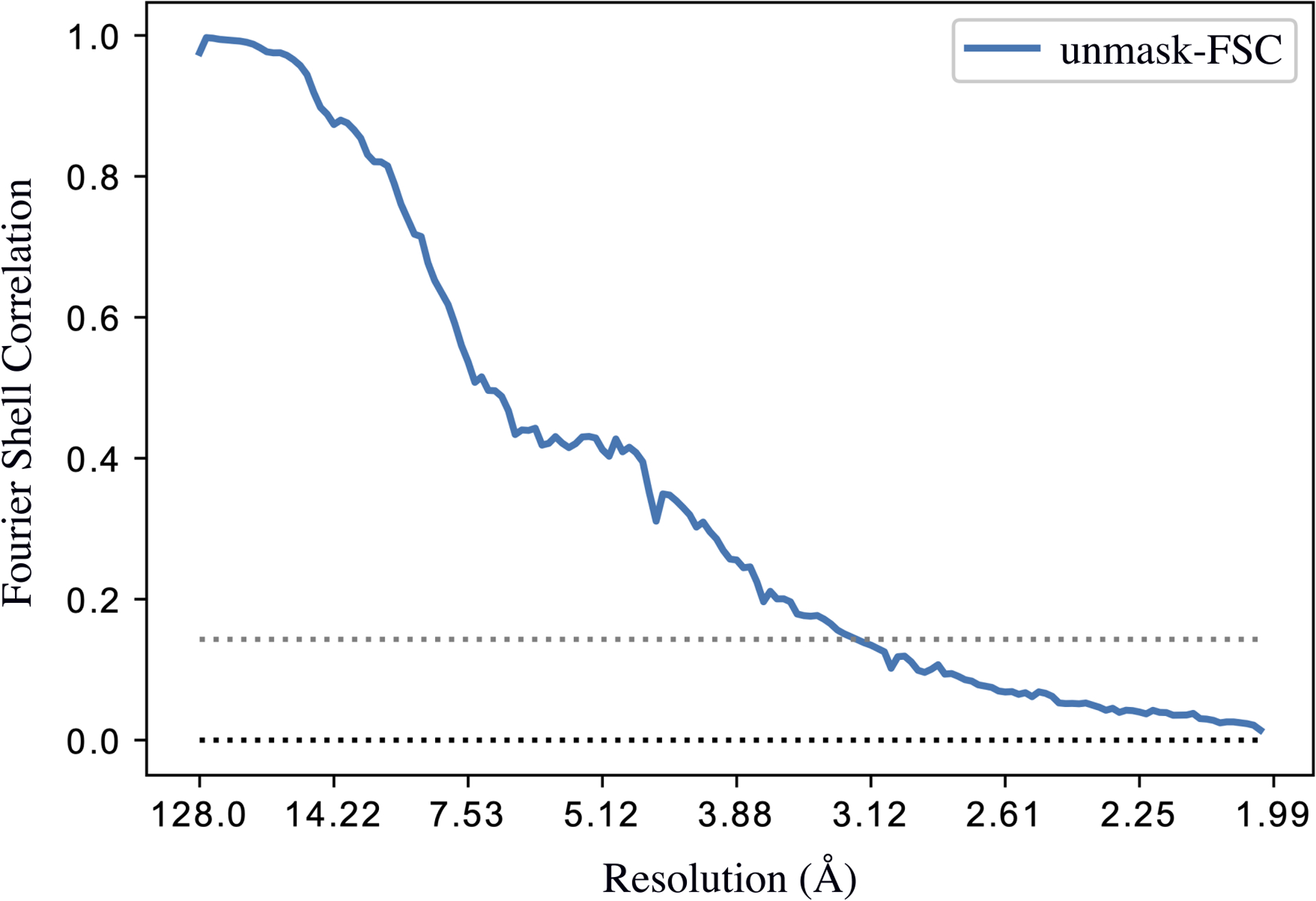
FSC curve comparing the state 05_10 input (ground-truth) and output (ESPER) 3D density maps. As one proceeds along the horizontal axis from the left (representing the center of the FT) to the right, increasingly larger shells are compared in Fourier space, such that the largest shells (far right) correspond to the highest resolution features. The FSC curve thus provides a global measure of how well one 3D density map matches the other. The upper dotted line indicates the threshold (0.143) used to determine the normal reproducible resolution [5].

We additionally validated our results by calculating the Fourier shell correlation (FSC) [5] between 3D density maps recovered by the ESPER method and their ground-truth counterparts (Fig. 10), and found a good agreement of all states up to a resolution near 3 Å, the ground-truth value. Q-scores [53] were also used as a local quantitative validation of the structural fidelity of the ESPER outputs. Using this approach on the ground-truth atomic-coordinate structures and their corresponding ESPER-recovered 3D density maps, we found highly favorable agreement across all residues in each state. On average, the Q-scores obtained were approximately 1.3 times that of the expected value (i.e., the average Q-score at a resolution of 3 Å), as calculated based on a data bank of reported resolutions of 3D cryo-EM density maps [53].

**Fig. 11.**
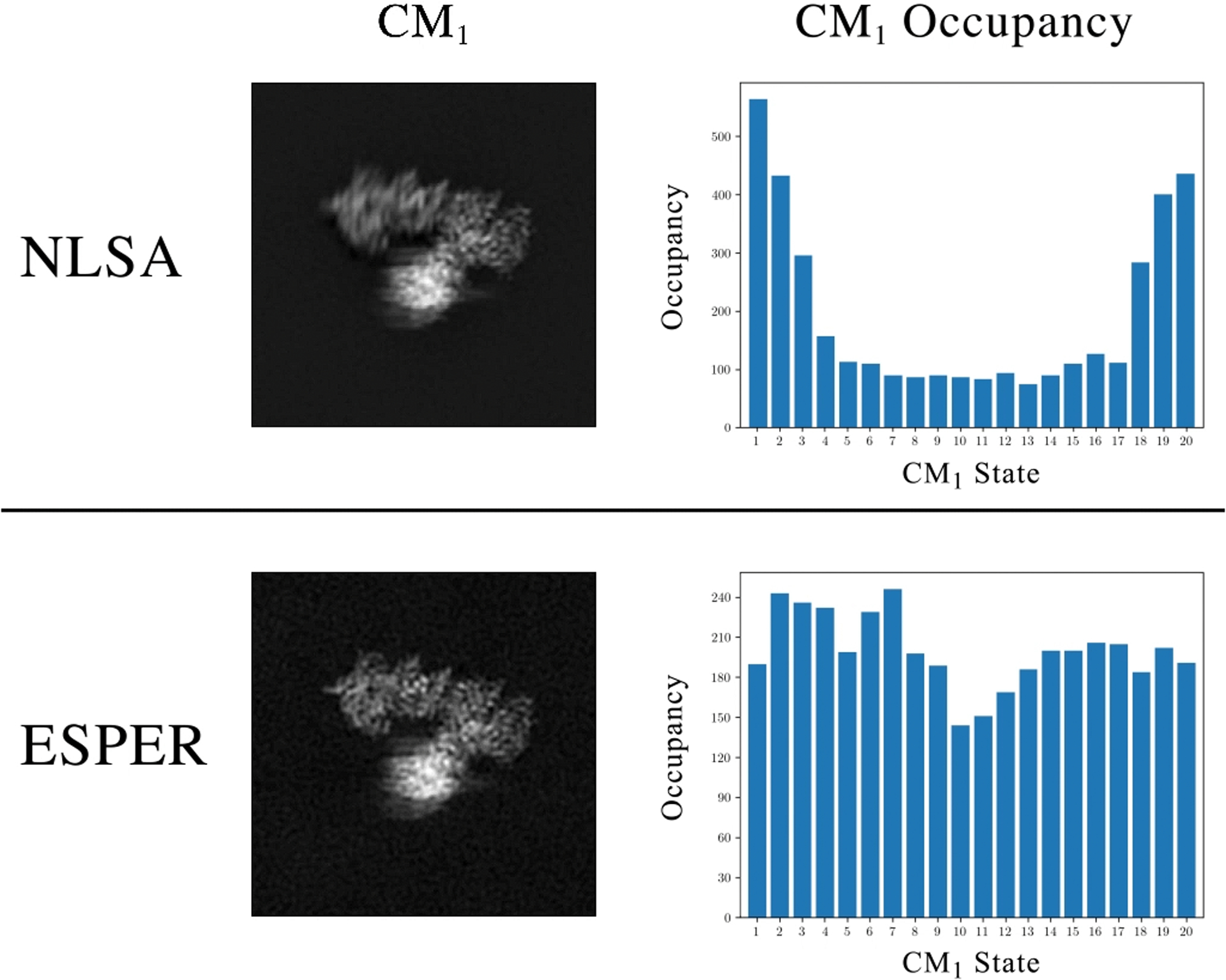
Snapshot taken from Movie 9, showing the stark difference in resolution between the Hsp90 arm motion (CM_1_, top-most domain) reconstructed by NLSA and ESPER. Occupancy assignments are also compared, showing an approximate bimodal (i.e., correct) distribution for the ESPER trajectory. An approximation of this kind is not available via the NLSA occupancy assignments, which also include serious problems near the boundaries.

A comparison of the outputs of ManifoldEM using either the ESPER or NLSA route for three example PDs from data-type IV are provided in Movie 9 (with a snapshot shown in Fig. 11), with these PDs selected based on both the visual appearance of their images and their embedded geometries. It should be noted that the same preliminary steps were performed for both methods (i.e., steps 1 and 2 in Fig. 8) before the branch in the workflow. During this branch, recall that the ESPER method includes the use of a unique 2D subspace per CM, while avoiding parabolic harmonics and applying eigenfunction realignments, ultimately resulting in 2D and 3D movies that retain the raw cryo-EM images. In contrast, the NLSA approach operates on only one of the initial DM eigenvectors per CM, and performs no steps for avoiding eigenvectors or realigning subspaces. In the process, the raw cryo-EM images are unavoidably discarded during the NLSA procedure, ultimately resulting in final 2D and 3D movies formed from NLSA-interpolated images.

Immediately apparent for all three PDs in Movie 9 is the difference in quality of the Hsp90 domains under motion corresponding to the given CM. For ESPER, these domains are highly resolved across all frames produced, while for NLSA these regions are much less resolved and noticeably smeared out. Second, while the visual differences between frames of the ESPER movies appear to evolve at an even pace, differences in frames appear less emphasized near the beginning and end of the NLSA movies, as if the movie was decelerating near these regions. In addition, the NLSA occupancies share little resemblance to our ground truth, with errors accentuated near the boundaries. Similar boundary problems do exist but are significantly less pronounced in the ESPER-derived occupancy maps, with each map showing reasonable agreement with ground truth (i.e., bimodal for CM_1_ and unimodal for CM_2_).

Differences in outputs due to methodology are most pronounced for the example PD_33_, which is a representative from the class of embeddings with appreciably unaligned eigenfunctions from the ideal eigenbasis, with the subspace of CM_2_ here requiring a larger counter-rotation than CM_1_. As can be seen, the overall range of motion for CM_1_ is noticeably reduced compared to outputs from ESPER. For CM_2_, matters are much worse. While our procedure using ESPER correctly charted a rotated, properly-aligned set of eigenfunctions, ManifoldEM employing NLSA used the existing embedding without accounting for realignment. As a result, the 2D movie produced by the NLSA method having closest resemblance to CM_2_ (i.e., Ψ_4_) demonstrated a physically-impossible sequence of motions: the splitting of the CM_2_ domain into two separate domains. At the end of Movie 9, the NLSA 2D movies obtained for the leading four eigenvectors are shown for comparison. Here, both (*i*) a physically-impossible splitting of the CM_1_ domain, and (*ii*) a subdued CM_2_ motion can be seen in the 2D movies obtained for both Ψ_2_ and Ψ_3_. A more detailed account of this comparison is also available [41].

In summary, while the NLSA and ESPER methods have operated on the exact same data—even up to generation of identical manifold embeddings—only ESPER is able to fully leverage the geometric structure present to consistently recapitulate ground-truth CMs and occupancies from a variety of PD manifolds. Further, while the ESPER method offers strategies to procedurally avoid introduction of nonsensical contextual output, NLSA can generate 2D movies with a wide range of defects [35], [36], [38], with each having the potential of appearing as a likely CM candidate to the naïve eye.

Finally, we note the total computation time for performing these two techniques on the same CM-eigenvector (Ψ_1_) from PD_2_, with final output a single 2D movie (as seen in Movie 9). While the application of the ESPER method to retrieve a 2D movie required approximately three minutes, the total computation time for NLSA for this same endeavor was over 90 times longer, with both methods having been run using a single-processor on the same workstation (3.8 GHz 8-Core Intel Core i7; 8 GB 2667 MHz DDR4). We additionally note that in the current release of the ManifoldEM framework [35], [36], it is required that this time-expensive NLSA computation is repeated in its entirety for every Ω_PD_ eigenvector chosen during final compilation of the free-energy landscape. Meanwhile, applying our intersection of image-indices approach—as afforded by retainment of the raw cryo-EM images—the ESPER method compiles CM content for all PDs and generates the free-energy landscape within minutes. All in all, the ESPER method has the potential to push the total computation time for a typical data set of approximately 500,000 images down from weeks or months to only a few days.

These high computational demands were rationalized for the implementation of NLSA as a way to handle unknown manifold structures [1]. In contrast, our heuristic analysis directly informs us of anticipated characteristics of the spectral geometry, enabling us to circumvent these previous unknowns, and perform the necessary operations required to accurately retrieve high-resolution images and a corresponding occupancy map for all CM states. Based on this knowledge, the ESPER method is able to produce appreciably more accurate outputs than the previous technique in a fraction of the time.

## VI. Results With Experimental Data

To assess the performance of ESPER—and the capacity of our heuristic knowledge—on real, experimentally-obtained data, we deploy our method on two data sets: the 80S ribosome from yeast [1] and ryanodine receptor type 1 (RyR1, *ligand-free)* [54]; both of which have been previously studied using ManifoldEM with NLSA [1], [24]. As these data sets are used only to compare outputs using either ESPER or NLSA, minimal conclusions will be supplied pertaining to the biological context of the results. Descriptions of experimental details are available in Supplementary Section F.

Motivated by our analysis of the synthetic data, we first searched through the experimental data sets for Ω_PD_ embeddings with distinct geometric features matching those encountered during our ground-truth studies. This search was enabled by the interactive tools in the ManifoldEM Python GUI [36], which provides a flexible means to view the distribution of images and occupancy of each PD as the angular width of each PD is uniformly altered. For different PD angular widths, up to 10° on *S*^2^, we embedded a set of highest-occupancy PDs and analyzed the results. Overall, the structure of all Ω_PD_ embeddings observed across these data sets fell into three broad categories, with leading subspaces exhibiting either a (*i*) *robust*; (*ii*) *marginal*; or (*iii*) featureless, *globular* geometric form.

For the majority of manifolds analyzed, embeddings with globular form were the most frequently encountered, followed by marginal, then robust. We found that the presence of each could be reliably predicted based on two parameters: the angular width and occupancy of the respective PD. Specifically, for PDs with small angular widths (approximately 3°) and a relatively high occupancy (typically greater than 1000 images), robust parabolic features emerged in the corresponding embedded subspaces.

Of the two experimental data sets, only the 80S ribosome was able to meet this criterion (and consistently for numerous PDs), which was statistically favored given the sheer number of images available: nearly 850,000 total. For the approximately 350,000 RyR1 images, PDs formed with 3° angular widths typically contained less than 400 images each, resulting in globular-shaped embeddings as expected from the results in Fig. 3. In the case of the RyR1 data set, as the angular width was increased to include a sufficient number of images in each PD, embeddings with marginal parabolic features emerged. Even still, these were intermixed with other PD embeddings exhibiting no apparent geometric features, which we assume is indicative of the presence of compounding factors, including: alignment mis-estimations, aberrant particles, and dispersed arrangement of angular assignments in a given PD. Further, since a vastly different manifold [19] (i.e., not a hypercube) is formed for images distributed on *S*^2^, the spectral geometry corresponding to a set of images falling within increasingly larger aperture widths, compared to a single point on *S*^2^, will become less marked by the features of a hypercube. Given this initial assessment, we next provide the outputs of ESPER on example subspaces exhibiting each of the three geometric properties observed.

### 80S Ribosome.

**Fig. 12.**
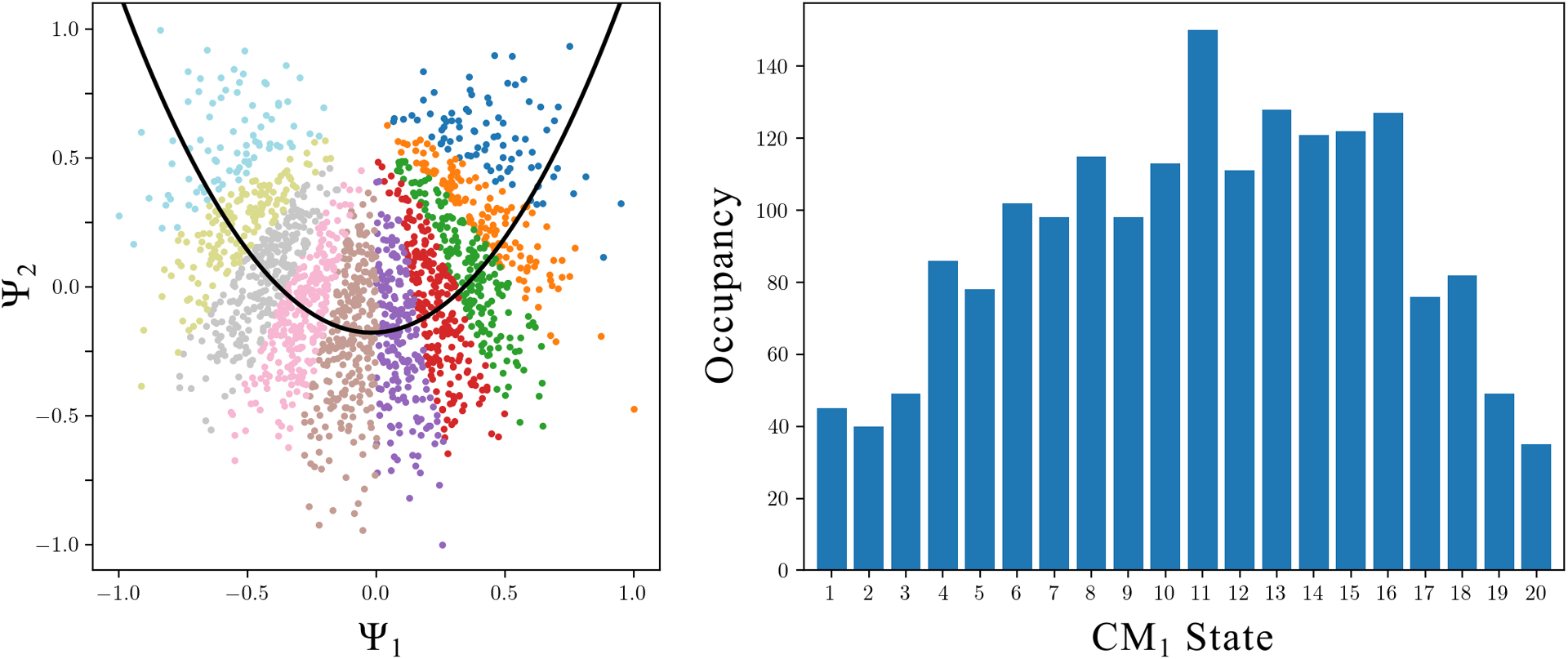
Results of applying ESPER on a robust parabolic point cloud obtained from the ribosomal data set. The corresponding PD was formed with a 2° angular width, containing a total of 1825 images.

The results of applying subspace partitioning (Algorithm 3) via ESPER on an embedded subspace with robust parabolic features is shown in Fig. 12. Since robust parabolic features were present only in the leading subspace {Ψ_1_ × Ψ_2_}, and given appropriate use of ${ℛ}_{\min}^{2}$ in Algorithm 1, eigenfunction realignment was not applied. To note, after defining CM_1_ at {Ψ_1_ × Ψ_2_}, Algorithm 1 additionally defined all eigenvectors Ψ_*j*_ in composite with Ψ_2_ (i.e., {Ψ_*i*_ × Ψ_*j*_}) as CM_1_ harmonics. The 2D movie results for the first four eigenvectors are provided in Movie 10, with outputs using ESPER compared directly with those obtained from the same Ω_PD_ embedding using NLSA. At the end of Movie 10, a schematic of the 80S ribosome is shown as viewed from this PD, with domains labelled.

The 2D movies produced by NLSA align well with the findings described in the original analysis [1], which were obtained from a suitable great circle in that analysis, where “typical” embeddings showed a parabolic form. The 2D NLSA movie we obtained corresponding to Ψ_1_ appears to exhibit the previously-described CM_1_ as seen from the current PD, including a ratchet-like intersubunit motion, a closing of the L1 stalk towards the intersubunit space, and a rotation of small subunit head along its long axis [1]. The 2D movie corresponding to Ψ_2_ likewise appears to describe CM_2_ : a so-called nodding motion of the head which is needed for selection of tRNA during decoding [1]. However, this motion is not isolated, and is also accompanied by similar—yet more subtle—domain motions as seen in the Ψ_1_ NLSA movie. The third NLSA movie (Ψ_3_) exhibits a collection of subtle domain motions as seen in the first two NLSA movies, while in the fourth NLSA movie (Ψ_4_), there appears to be a previously-undescribed shift in intensity within the intersubunit space. Through NLSA, the original work [1] defines the {Ψ_1_ × Ψ_2_} subspace as the basis for constructing the 2D energy landscape, corresponding to the motions observed in both the Ψ_1_ and Ψ_2_ NLSA movies.

As seen in Movie 10, the ESPER method provides nearly identical outputs as achieved by NLSA for Ψ_2_, Ψ_3_ and Ψ_4_. (To note, we had to force the calculation of Ψ_2_ in the ESPER workflow, as it was initially removed as a harmonic in Algorithm 1). A striking difference appears, however, in the sequence of states describing the leading CM, and specifically as it pertains to the trajectory of the head subunit. Instead of a simple left-to-right head motion as seen via NLSA, the motion charted by our method shares a likeness to a combination of the motions observed in both the first and second NLSA movies. Specifically, in the ESPER movie, the head subunit is first seen moving up and to the left, followed by a downwards motion (nodding) into the intersubunit space. Meanwhile, all other domains charted for CM_1_ by the ESPER method move in a consistent fashion to those seen in the Ψ_1_ NLSA movie.

As understood in our approach, and in contrast to the analysis performed using NLSA, the {Ψ_1_ × Ψ_2_} subspace is fundamentally not a 2D state space {CM_1_, CM_2_} laid out conspicuously like a parabola, but actually a parabolic point-cloud $\left\{\psi_{1}^{1}\times \psi_{2}^{1}\right\}$ representing a single degree of freedom (CM_1_). Accordingly, our analysis calls into question the authenticity of the NLSA-interpolated motion along the base of the triangular path in the original 2D landscape [1]. This noticeable difference in interpretation arises strictly due to the different treatments of subspace geometry by the two methods. While NLSA projects onto one eigenvector before organizing images into supervectors to form interpolated NLSA movies, the ESPER method carefully charts the geometric structure of the 2D subspace, and creates each frame of the respective movie by selectively averaging subsets of the available cryo-EM images.

### Ryanodine receptor (RyR1).

We next describe the results of ESPER on embeddings with marginal and globular geometric structure. In Movie 11, we show the 2D movies obtained from an RyR1 PD containing 976 images within an angular diameter of 6°. Here, the {Ψ_1_ × Ψ_3_} point cloud corresponding to CM_1_ has a significantly more marginal parabolic appearance compared to those observed for the ribosome, while the best-fit point cloud for CM_2_ at {Ψ_2_ × Ψ_4_} appeared devoid of parabolic features al-together. For this embedding, the conformational motions output by using ESPER for each 2D subspace were highly similar—albeit noisier—to those output by NLSA for each eigenvector individually. In either case, the leading CM corresponds to the entire assembly of cytoplasmic shell, activation core and pore (appearing like an opening of the central channel pore), while CM_2_ corresponds to movement of the cytoplasmic shell resulting in an apparent lowering of the handle and clamp domains.

Overall, these results are comparable to those described in the original study [24], with no major deviations observed, other than noise, between the performance of NLSA and ESPER. For this latter discrepancy, we found that by noise-filtering each 2D movie produced by ESPER using singular value decomposition (as denoted in Movie 11 with the initials “SVD”), we were able to very closely reproduce the appearance of the NLSA outputs (which, recall, are intrinsically noise-reduced). This likeness increases with the occupancy of the PD, which corresponds to the presence of more pronounced signal in the initial 2D movie used for decomposition during SVD. As a loose estimate, below 900 images we begin to see a significant drop in the consistency of these SVD outputs.

## VIII. Discussion

The findings in this study are based on heuristic information obtained from simulated, controlled data sets which we have thoroughly analyzed to formulate a method—termed ESPER—able to accurately and efficiently recapitulate ground-truth information. Specifically, we have identified the way sets of images originating from a molecular machine’s varying atomic structure are represented in low-dimensional embeddings obtained by prominent dimensionality-reduction techniques, and how to navigate this spectral geometry to recover the machine’s conformational continuum. Our findings on synthetic data sets—which encompass multiple degrees of freedom, nonuniform occupancy maps, and experimentally-relevant noise and CTF—provide a number of new insights unaccounted for in the founding ManifoldEM framework [1].

We additionally introduced alternative methods for producing synthetic data, which were used to create the Hsp90 and the mouth-wings continua. The latter example, which includes complex conformational changes that go beyond rotation of domains, illustrates the broader scope of our heuristic analysis, which not only provides insights for cryo-EM data, but also for projection data obtained through other methods of visualization. Several portions of our more-detailed heuristic analysis have also been directly extended to other experimental techniques dealing with alternative manifold inputs, such as the use of atomic coordinates in molecular dynamics and 3D density maps in cryo-electron tomography [41]. As such, we believe that there is a potential for the application of these insights to a wide range of experimental data sets beyond cryo-EM, and particularly those obtained from systems exercising multiple degrees of freedom in a continuous manner.

By applying the ManifoldEM framework on our synthetic data, we demonstrate that serious problems can emerge in the analysis, including presence of physically-impossible, stunted, or hybrid conformational motions (CMs), as well as erroneous occupancy maps. These issues mainly arise during one critical step, where the geometry of each embedding must be correctly charted to render a set of CMs and corresponding occupancies. This task is originally performed in most part by the application of NLSA [33], where each eigenvector of the diffusion map (DM) embedding is treated as an independent coordinate for a conformational change. By concatenating cryo-EM images along a given eigenvector, interpolated images are produced via NLSA, and re-embedded to form a new space from which a 2D NLSA movie is extracted representing the deduced CM.

However, our heuristic analysis shows that the observed problems can arise when each eigenvector is treated as an independent source for a CM, while in actuality, a single eigenvector can correspond to some combination of CM eigenfunctions, as well as to eigenfunction harmonics. We additionally demonstrate how each CM is better mapped by a parabolic trajectory in a 2D subspace defined by two corrected eigenvectors, for which the projection of that trajectory onto a single eigenvector is naturally problematic. Our analysis found that it was essential to correct for these properties in order to accurately map each CM. Depending on the PD and data characteristics, these issues can combine to create several systematic errors, limitations and uncertainties that were previously pointed out [35], [36], [38].

We have developed the ESPER method as a means to circumvent these problems and refine the existing framework. The operations introduced in this study offer several enhancements, including our procedure for isolating CM subspaces, removing CM harmonics, correcting for eigenfunction misalignments, and directly retrieving each CM from the raw cryo-EM snapshots as arranged within the initial (corrected) embedding. In the last case, the use of the raw images is shown to improve both the accuracy of occupancy determination and final resolution of 3D structures, while providing a vastly simplified workflow for determining multidimensional free-energy landscapes. We have further shown that our implementation of these enhancements drastically decreases the overall computation cost.

All of this said, the ESPER method is not without its own limitations and uncertainties, which are least pronounced for relatively well-behaved synthetic data. Despite its remarkable performance on our 126-PD data set with experimentally-relevant SNR and CTF, we believe there is still room for improvement of our eigenfunction realignment technique. Specifically, future works could aim to deal with complex physical constraints (e.g., due to steric hindrance between moving domains) as well as data sets with a larger number of degrees of freedom. In the former case, the use of additional rotation operators may be required [41], creating a more complex tree of decisions, with ESPER outputs possibly refined by a maximum-likelihood approach or by using a neural network. Furthermore, for noisier, less-structured embeddings, the 2D histogram method may provide suboptimal counter-rotations. Besides, more robust procedures should be employed to identify and fit both highly-structured and less-structured regimes, such as a constrained least squares method [55] or a generalized Hough transform [56].

With the use of synthetic data, we also show that final occupancy assignments can have slight inaccuracies, which are most emphasized near the boundaries of each CM. Although not pursued here, since our method retains the raw cryo-EM images, these misassignments could be further corrected to improve 3D density maps and corresponding occupancy distributions. Specifically, an optimization approach could be designed to compare images within each bin and reassign erroneously-assigned snapshots into neighboring bins in which they most likely belong. To note, a maximum-likelihood approach does already exist that aims to extract such granular conformational heterogeneity [42], as does a method based on neural networks [32]. A more comprehensive discussion of additional, less-impactful improvements to the core ESPER method is also available [41].

Our findings on both synthetic and experimental data sets establish a minimum requirement for PD-manifold studies of conformational continuum. Specifically, we have found that for maximal fidelity of final outputs with ground truth, a data set must contain well-structured geometry when embedded. The performance of ESPER hinges on the presence of such geometric information, and as the quality of the embedded geometry increases, more of our method’s benefits become available. As seen in our two examples, the ability to sensibly avoid harmonics or apply eigenvector rotations is only applicable up to the number of CMs present having pronounced geometric structure that is viable for reliable parabolic fits. If no geometric form can be deciphered in a PD embedding, it is effectively impossible to solve for these unknowns. Further, upstream errors in angular assignments will only worsen these trends and, depending on the severity of the error, critically undermine the efficacy of the ManifoldEM framework [38]. These misalignments present an unavoidable conflict that can only be addressed at the source.

This limitation presents a problem when dealing with typical experimental data sets, where it is most realistic to anticipate the presence of only a subset of PD embeddings which have adequate geometric information as required by the ESPER approach. Even given just one such high-quality PD, our analysis shows that the ESPER method is able to provide essential information on the molecular machine’s conformational spectrum. If such a PD-embedding was both highly-populated and available from a viewing angle where all CMs were well-visualized, all information pertaining to the machine’s total number and approximate types of degrees of freedom—as well as corresponding occupancies—could be accurately calculated from the images of a single PD alone.

We next expand this idea to the more likely presence of a subpopulation of such informative PDs, with the ESPER approach individually applied on each. To reconstruct adequate 3D density maps, alternative methods would then need to be devised to effectively fill in conformational information for the remaining PDs lacking geometric form. Indeed, the reliability of our approach decreases rapidly when approaching the regime of globular embeddings, since there is no geometric information to leverage. For these remaining PDs, the NLSA approach is better suited, since it can at least retrieve reasonable 2D movies from low-occupancy embeddings. However, since the apparent absence of geometric features in these embeddings does not discount their latent presence and potential impact, NLSA outputs may still incur the known limitations and uncertainties [35], [36], [38]. To mitigate these unavoidable issues, we recommend an altered use of NLSA, which is directly informed by the conformational spectra obtained by the ESPER method in the highest-quality PDs. Under such a scheme, the ESPER outputs would serve as a high-quality template on which NLSA outputs can be mapped.

In this tradeoff, there exists some gray area where it is difficult to make out which technique should take precedence. Certainly, low-occupancy globular embeddings should be handled by NLSA, and although the ESPER outputs on high-occupancy globular embeddings are similarly convincing and highly consistent with NLSA, it is our belief that the decision to run ESPER over NLSA on a given PD should be governed by a sensible coefficient of determination threshold ${ℛ}_{\min}^{2}$. Specifically, for each Ψ_*i*_ the fit score ℛ^2^ corresponding to the 2D subspace with the highest fit score among all other {Ψ_*i*_ × Ψ_*j*_} subspaces should exceed the value of ${ℛ}_{\min}^{2}$. For embeddings with fit scores above this threshold, the ESPER method can leverage a number of benefits over NLSA, with this number increasing as the quality of the geometry improves. Since the appearance of robust geometry is also dependent on high PD occupancy (Fig. 3), and high PD occupancy boosts signal, the ESPER method is additionally qualified to furnish high-quality SVD movies in this regime while retaining the raw cryo-EM images.

As we have shown for RyR1, these noise-filtered 2D movies have a quality almost identical to the respective NLSA outputs, and serve the single purpose for use by belief propagation across *S*^2^ during CM assignments. In noisier circumstances, SVD results can be enhanced by binning the movie frames to boost signal. (It is also worth investigating alternative methods for these means). Meanwhile, the raw cryo-EM images are retained for use during 3D reconstruction, and, aside from improving fidelity of those outputs, allow use of our efficient approach using intersection of image-indices in generating occupancy maps and energy landscapes. Since our proposed strategy leaves the PD-embeddings without discernable geometry to be analyzed using the preexisting NLSA approach, final outputs would next need to be combined between these two methods. Notably, for each PD analyzed by either ESPER or NLSA, the corresponding free-energy landscape and projections (i.e., raw cryo-EM images or NLSA images, respectively) must be combined to form a consolidated free-energy landscape and corresponding 3D density maps. If necessary, SVD could then be applied on the final sequence of reconstructed 3D density maps, as has previously been done in ManifoldEM [35], [36]. We anticipate that the next public distribution of the ManifoldEM Python suite [36] will include these advancements as a significant refinement to its workflow. Finally, we hope that the insights gained from these machine-learning heuristics on image ensembles will be useful not only in the cryo-EM field, but more broadly to other methods dealing with projection data, as well as to the general development of techniques aimed at untangling complex systems exercising multiple, continuous degrees of freedom.

## Supplementary Material

Supplementary Material

Movie 1

Movie 2

Movie 3

Movie 4

Movie 5

Movie 6

Movie 7

Movie 8

Movie 9

Movie 10

Movie 11
