# Supplementary material for: Recovery of Conformational Continuum From Single-Particle Cryo-EM Images: Optimization of ManifoldEM Informed by Ground Truth
Source: IEEE Trans Comput Imaging. Author manuscript; Available in PMC 2022 Oct 17. (PMC9575687; doi:10.1109/tci.2022.3174801)
Supplement: Supplementary Material [file NIHMS1839740-supplement-Supplementary_Material.pdf]

Supplementary Materials

A. Simulation of Hsp90 Atomic Models

This section provides essential information on the construction of our synthetic data, with more detailed documentation [1] and a repository of code for generating custom data sets [2, 3] available in archival depositions online. Additional to the code in [2], our IEEE DataPort repository [3] includes all Python scripts for reproducing the ESPER workflow, as well as the full Hsp90 dataset used in our final analysis (termed data-type IV in this document).

As a basis for simulation of ground truth data we chose heat shock protein 90 (Hsp90), which operates with two arm-like domains (chain A and B, containing 677 residues each) connected together in an overarching V-shape. *In vivo*, these arms are known to close after binding of the molecule with ATP, with Hsp90 acting as a chaperone to stabilize the structures of surrounding heat-vulnerable proteins. During its work cycle, Hsp90 naturally undergoes large conformational changes, transitioning from its two arms spread open in a full V-shape (inactive state) to both arms bound together along the protein's central line of two-fold symmetry (active state) following ATP binding.

Casting Hsp90's biological context aside, liberties were taken in the choice of the synthetic model's three conformational motions (CM_1, CM_2 and CM_3), each corresponding to a fully-decoupled domain motion. Each CM was designed to cover a unique range of motions, with the cascade of overlaid states making up CM_1 occupying the largest spatial region, followed in magnitude by CM_2 and then by CM_3. Using combinations of these CMs, three synthetic state spaces (SS_n) were generated, with intrinsic dimensionalities of $n = 1$, 2, 3. This was achieved by changing the positions of the first, the first two, or all three regions defined as rigid domains in their given ranges monotonically and—in the latter two cases—independently. In Figure 13, three states each are shown of Hsp90 exercising its domains according to either CM_1 or CM_2.

In our analysis, these state spaces are termed SS_1, SS_2 and SS_3, and contain: (*i*) 20 states exhibiting one degree of freedom (CM_1); (*ii*) 400 states (20×20) with two degrees of freedom (CM_1, CM_2); and (*iii*) 1000 states (10×10×10) with three degrees of freedom (CM_1, CM_2, CM_3), respectively. As a specific example of the ranges of motion present in SS_3, the Root-Mean-Square Deviation (RMSD) was calculated for the differences of the atomic coordinates between neighboring states in each CM, yielding the values of 1.8 Å, 1.3 Å and 0.3 Å along CM_1, CM_2 and CM_3, respectively; with the RMSD between the first and last state of each CM (representing the total span) yielding 15.3 Å, 11.3 Å and 2.4 Å.

In the following, we provide a description of the atomic-coordinate displacements between states for the specific case of our Hsp90 model exercising two conformational motions (CM_1 and CM_2), as seen in Figure 13. These CMs were designed to be fully decoupled from each other, with no mutual steric hindrance between domains within the ranges of their motions, nor energy minimization performed.

Atomic manipulations of the original PDB coordinate file were done using PyMOL [4]. For CM_1, the chain A arm was rotated outwards (directly away from chain B) from its central hinge at residue 677, in 1° increments until a series of 20 rotational states were defined. For each of these 20 CM_1 states, all residues above chain B's elbow (residues 12-442) were then rotated perpendicularly to the CM_1 motions in 2° increments until a series of 20 rotational states were defined for CM_2. These operations resulted in the creation of a total of 400 unique conformational states. The ensemble of these states can be organized in a 20×20 state space (SS_2), where each entry represents one of the possible combinations of CM_1 and CM_2. This state space covers our synthetic model's complete ensemble of physically allowable conformations (i.e., a quasi-continuum).

These 400 structures represented by PDB files were then each transformed into 3D electron density maps (EDMs) with a

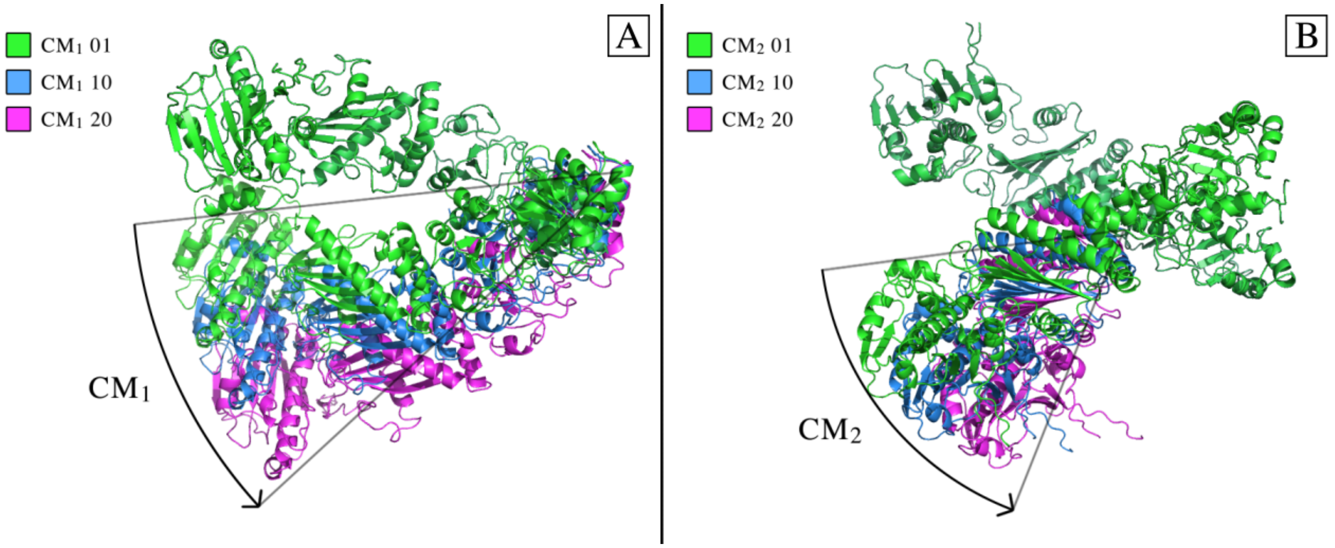

Fig. 13. Cartoon representations of Hsp90 atomic-coordinate structures displaying two CMs. In figure [A], a comparison of the CM_1 first, middle, and final state is shown. Similarly, in figure [B], a comparison of the CM_2 first, middle, and final state is shown.

sampling rate of 1 Å per voxel and simulated resolution of 3 Å using the EMAN2 [5] module “e2pdb2mrc”, where each atom is represented by a Gaussian with radius defined by the appropriate number of electrons. In reality, cryo-EM data are projections of the Coulomb potential distribution, which is distinct from the electron density distribution produced by X-rays. For the present analysis, however, this distinction is irrelevant. Projections of each EDM can then be obtained via standard parallel line integrals along the direction of the electron beam so as to simulate images generated in a transmission electron microscope (TEM) operated in the bright-field mode [6]. An animation cycling through the 400 SS_2 states as represented by the Hsp90 atomic structures has been provided at the top of Movie 1, with the ordering of states following a second-hand (CM_2) to minute-hand (CM_1) “clockwise” analogy.

B. Simulation of Mouth-Wings Model

In this section, we describe the creation of an alternative data set, the so-called “mouth-wings” model, that is entirely separate from the previously-detailed procedure used to generate each Hsp90 continuum. In comparison, this workflow provides a radically different approach to generating projection data, and is able to account for complex interactions within each domain motion. Importantly, the mouth-wings model is used only to demonstrate the generality of our approach; for all other purposes in our analysis, Hsp90 is used. With that said, we first introduce the mouth-wings toy model with a schematic shown in Figure 14-A.

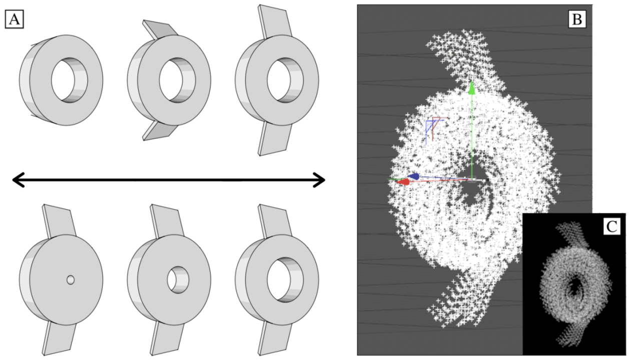

Fig. 14. Mouth-wings toy model capable of independently exercising two conformational motions: (i) opening and closing of the central “mouth”; and (ii) flapping of “wings”. The underlying state space is described by all pairwise combinations of these 20×20 domain motions, such that 100 states are realized in total.

Each conformation in the mouth-wings continuum is modeled and rendered using professional 3D modeling software (Cinema4D; [7]). To uphold the conservation of mass across all states, an ensemble of 2-spheres (loosely representing atoms) is generated in three arrays using the “cloner object”: (i) a cylindrical array with adjustable inner radius (representing the main body of the object, with its central opening termed the “mouth”); and (ii, iii) two thin, rectangular arrays on either of its sides (termed the “wings”). The spheres making up each section are positioned by hand (keyframed) to uniquely reposition across each range of their respective collective motion. Notably, the 2-spheres making up the “mouth” domain were keyframed for each state so as to gradually clump together, ultimately presenting a higher density towards the fully open state (Movie 2). In a similar vein, the “wings” were programmed in unison to both open at constant angle about their hinge, while also curling inwards.

In this way, 20 “mouth” and 20 “wings” states are defined, using all combinations to generate the 20×20 state space (SS_2). Next, via the “PyroCluster” plugin, the spheres are transformed into “thinking particles” (Figure 14-B), where each particle can be envisioned as a “puff” that, when rendered, effectively stacks with integrated intensity with all other particles present along the camera’s line of sight (Figure 14-C). A tessellated sphere is then generated by segmenting an icosahedron, with an orthographic camera assigned to each of its vertices to obtain projections of all states across 980 PDs spaced approximately 5° apart. An animation of these motions as seen from different viewing angles can be found in Movie 2.

C. Simulation of Experimental Conditions

The core framework in Section A is used as a basis for the creation of images that more closely approximate anticipated experimental conditions. These conditions are incrementally incorporated in four steps. Specifically, we use the term data-type I to refer to one copy of each state with pure signal and no CTF. Data-type II refers to uniformly-duplicated states with experimentally-relevant SNR, and, in addition to SNR, data-type III further introduces experimentally-relevant CTF. For our final analysis, data-type IV is constructed in the same way as data-type III, then given a nonuniform occupancy map.

Details for Data-type I. In the pristine scenario, for each EDM within a chosen state space (SS_n), sets of images in five PDs are first obtained. The first of these PDs was chosen to be normal to the plane of the CM_1 rotation, such that all CM_1 motions from that perspective only underwent changes in the plane of the projection. A similar choice was made for PD_2 for CM_2 motions, with the remaining PDs chosen at arbitrary positions in angular space. All projections were generated using the EMAN2 module “e2project3d” with no CTF applied. For each state space, the set of these images as generated from one of these five PDs forms a high-dimensional manifold Ω_{PD}. For the case of SS_2, this procedure resulted in the creation of 400×5 unique 2D images for each of our 400 3D EDMs. Movie 1 provides an animation of Hsp90 exercising through all of the SS_2 conformations in a clockwise sequence, as viewed in each of these five PDs.

Details for Data-type II. In this scenario, images generated for data-type I are used as a base, from which they are then uniformly duplicated τ times. Additive Gaussian noise is next applied to each image individually to grant it a specific SNR that is the same for all images in the set. We define the SNR by the ratio of each image’s signal variance to its noise variance [6]. Here, signal represents the 2D region of pixels corresponding to the average area occupied by the macromolecule. This region is obtained by masking out all

pixels within one standard deviation of each image's mean intensity value, and in effect, excluding the approximately uniform-intensity background. This process was thus performed by first finding the mean pixel intensity (μ_{signal}) and variance of the signal (σ^2_{signal}), and then calculating

$$\sigma^2_{noise} = \sigma^2_{signal} \,/\, SNR. \tag{7}$$

Using this parameter, we then apply additive Gaussian noise to each image in order to obtain an output image having the desired SNR (Figure 15). In this process, a sample from the Gaussian distribution is added to each pixel's intensity. Each resulting image was then normalized such that the average pixel intensity and standard deviation of pixel intensities was approximately 0 and 1, respectively. As a note on experimental relevance, an SNR of 0.1 has been previously established as a suitable choice for experimental SNR in images obtained by cryo-EM [8].

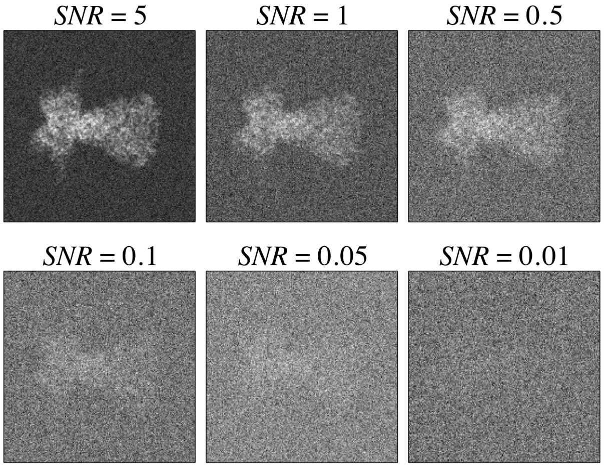

Fig. 15. SNR simulation on projections of Hsp90. Specifically, the first image in SS_1 is shown, from a PD with different values of SNR, as obtained via additive Gaussian noise with appropriate statistics.

Details for Data-type III. This scenario again uses images generated for data-type I as a base. Similar to data-type II, τ = 10 duplicates per state are then generated. However, instead of immediately applying additive Gaussian noise to each image, each image is first filtered by the TEM's CTF in an experimentally relevant range. For this task, CTF is generated via the form $H(k) = \sin\chi - A\cos\chi$, defined with

$$\chi = -\pi\Delta z\lambda k^2 + \tfrac{\pi}{2}\, C_s\, \lambda^3 k^4. \tag{8}$$

Here, Δz is a defocus value randomly assigned in the interval [5000, 15000] Å (positive is underfocus); λ is the wavelength of the electron as calculated via known TEM voltage; k is the spatial frequency; C_s is the spherical aberration; and A denotes the fraction of amplitude to phase contrast [6]. Once the CTF is generated with a choice of relevant microscopy parameters, it is next applied through scalar multiplication with the Fourier transform of the image, followed by an inverse Fourier transform of the product. After this procedure, additive Gaussian noise is uniquely applied to each image granting an SNR of 0.1. It should be noted that later, when CTF correction is performed, we use exactly the same CTF for initially modifying each image as we use for correcting it, thus making no allowance for experimental inaccuracy of CTF estimation.

Details for Data-type IV. For this data set, instead of a constant τ, an arbitrary nonuniform occupancy distribution was created for the SS_2 {CM_1, CM_2} map. (I.e., no attempt was made to link the occupancy map to the realistic energetics of the molecule simulated). In thermal equilibrium, differences in occupancy can be attributed to differences in the molecule's free energy ΔG, such that an occupancy map can be transformed into a free-energy landscape via the Boltzmann factor

$$\Delta G / k_B T = -\ln(n/n_0), \tag{9}$$

where n is the occupancy of the current state and n_0 is the occupancy of the maximum-occupancy state in the state space [9]. Here, the Boltzmann constant k_B is a physical constant that relates the average relative free energy of particles to their bulk temperature T. The characteristics of our simulated occupancy map were chosen to provide easily distinguishable features along both 1D and 2D CMs: specifically, bimodal and unimodal distributions for CM_1 and CM_2, respectively (Figure 16).

Finally, since data-type IV is used for an analysis of the final 3D reconstructions, a sufficient number of PDs is required. The minimum number of equispaced PDs (P_{min}) on a great circle required for 3D tomographic reconstruction at a given resolution is obtained from the Crowther criterion [10]

$$P_{min} = \pi D \,/\, r, \tag{10}$$

where D is the particle diameter (120 Å, as measured in state {20, 20} of SS_2) and r is the targeted resolution of the reconstructed volume (for our purposes, 3 Å was chosen to match the resolution of the ground-truth EDMs). According to this criterion, we generated 126 equidistant PDs spaced 1.5° apart along one half of a great circle, chosen so as to avoid redundant information due to diametric mirroring.

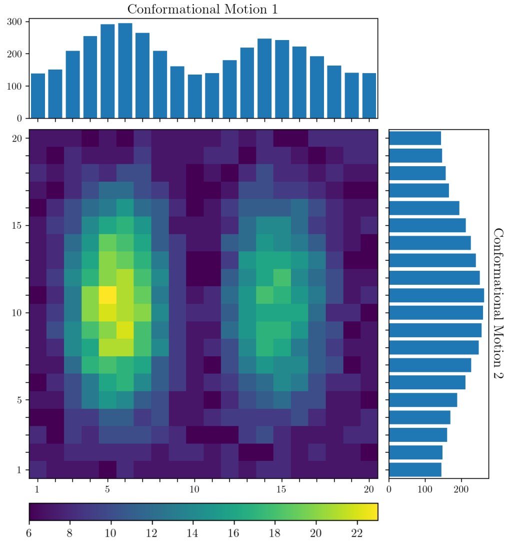

Fig. 16. 2D distribution of ground-truth occupancies for all 400 states in SS_2, assigned equally for each PD.

Each of the 400 SS_2 states in each of these 126 PDs was then duplicated based on assignments imposed from our arbitrary occupancy map (Figure 16), resulting in 4000 images per PD, with each image modified with an individual CTF having randomly-assigned defocus, as previously described. Finally, additive Gaussian noise (SNR of 0.1) was applied to each image such that 504,000 unique images were created in total.

D. Dimensionality Reduction Methods

In the following, a summary of both nonlinear (DM) and linear (PCA) dimensionality-reduction methods is provided as they relate to the embedding of cryo-EM images in each PD.

Diffusion Maps. Given a set of N images, the DM [11] approach seeks to generate an optimal embedding of the data in a low-dimensional space, so as to preserve all relevant information. We first normalize each of the images by removing the mean and scaling to unit variance. As the kernel required for the DM framework is formed using pairwise distances, we first create the distance matrix $\boldsymbol{D}$ (for each PD independently) by calculating the Euclidean distance [12] for every pairwise combination of its N images. The Euclidean distance between two images $X = (x_1, x_2, \ldots, x_P)$ and $Y = (y_1, y_2, \ldots, y_P)$, where x_i and y_i denote the intensities at pixel i in images X and Y (each having P pixels), is defined by

$$D_{X,Y} = \left(\sum_{i=1}^{P} (x_i - y_i)^2 \right)^{1/2}. \quad (11)$$

The pairwise distances form a symmetric $N \times N$ matrix, where a single row represents the distance of the corresponding row-indexed image to each column-indexed image. Next, an isotropic Gaussian kernel is applied to these distances to create a real, symmetric similarity matrix

$$A_{i,j} = \exp(-D_{ij}^2 / 2\varepsilon). \quad (12)$$

The similarity matrix $\boldsymbol{A}$, calculated using a suitable Gaussian bandwidth ε, is then divided by a diagonal matrix of its row sums to construct a symmetric, positive semidefinite stochastic Markov transition matrix $\boldsymbol{M}$. This matrix represents the relative pairwise affinity between all images and is closely related to the normalized graph Laplacian $\boldsymbol{L} = \boldsymbol{I} - \boldsymbol{M}$, with $\boldsymbol{I}$ the identity matrix [13]. Eigendecomposition of the matrix $\boldsymbol{M}$ is then performed to retrieve an ordered set of N eigenvalues and corresponding eigenvectors, which define a nonlinear spectral embedding of the data [14].

The Gaussian bandwidth (ε) in the above expression has a strong influence on the definition of similarity between the cryo-EM images. At small Gaussian bandwidths, the system takes on a relatively fine-grained definition of similarity, while increasing ε transforms this relationship into a more coarse-grained notion of similarity. These notions of similarity govern the geometric structure of the resultant manifold embedding. Particularly, in the limit $\varepsilon \to 0$ and $N \to \infty$, and with an appropriate normalization of the similarity matrix [11], the DM eigenvectors converge to the eigenfunctions of the LBO [14]. In all of our results presented, the DM embedding has been generated within its optimal range of Gaussian bandwidth.

Finally, we introduce here the *double-filtering* kernel established by Dashti *et al*. (2014) used to account for introduction of a different CTF on each cryo-EM image. If uncorrected for, images with different CTFs—as constructed in our study and gathered by design in an experiment—cannot be directly compared using a standard distance metric. The double-filtering kernel compensates for defocus differences by adjusting each pairwise Euclidean distance calculation. Specifically, during this calculation, the Fourier transform of each image is multiplied by the CTF of the image under comparison. This operation ensures a zero Euclidean distance between any two images that differ in defocus only [15].

Principal Component Analysis. Instead of defining the Gaussian kernel as previously used in the Markov transition matrix, PCA is performed on the data matrix Z with dimension $P \times N$). Before embedding, the images are normalized by removing the mean and scaling to unit variance. Eigendecomposition is next performed on the $N \times N$ matrix $Z^T Z$ to generate a set of orthogonal eigenvectors (i.e., principal components, PC) and corresponding eigenvalues.

To note an important comparison between PCA and DM, the matrix $Z^T Z$ is symmetric and positive semi-definite (i.e., all eigenvalues are non-negative [16]), which is also the case for the Markov transition matrix used in the DM framework.

E. Overview of NLSA

In the founding ManifoldEM framework [15], nonlinear Laplacian spectral analysis (NLSA) [17] is applied independently on each member of a leading set of Ω_{PD} eigenvectors in order to assess the "meaning" of each in terms of housing potential CMs of interest. For each of these eigenvectors, NLSA is performed as follows. First, the raw images are concatenated along a chosen eigenvector to produce so-called "supervectors" [15]. Nonlinear singular value decomposition (SVD) is then used to extract noise-reduced NLSA images ("topos") and their evolutions ("chronos") from these supervectors. These NLSA images are then embedded to form a new set of eigenvectors in a different space, which results to high accuracy in a 1-manifold with known eigenfunctions $\{\cos(k\pi\tau') \mid k \in \mathbb{Z}^+\}$, parameterized by a conformational parameter τ' (separate from the use of τ in our current work). This enables the estimation of the density of points as a function of τ' together with an ordered sequence of 2D NLSA images. These 2D images can be arranged to form a 2D NLSA movie, designed to represent the conformational signal corresponding to the eigenvector from the initially-embedded PD manifold. Once a set of 2D NLSA movies have been constructed along each of the leading Ω_{PD} eigenvectors independently, supervised identification of "meaningful" CM information is next required.

When knowledge of only one degree of freedom is desired (or available), the 2D movies corresponding to the same CM in different PD manifolds can be further compiled across S^2 to reconstruct NLSA volumes and thus a 3D movie representing the CM. The NLSA procedure is much more complicated when information of two (or more) degrees of freedom is desired.

After supervised identification of two CMs, their respective eigenvectors for the current Ω_{PD} are used to isolate a 2D subspace therein. On this $\{\text{CM}_1, \text{CM}_2\}$ subspace, NLSA is performed independently along the directions of (typically) 180 uniformly-spaced radial lines in the range $\theta \in [0, \pi]$. This yields a collection of point densities (i.e., 1D occupancy maps) $n(\tau', \theta)$ for each θ. The collection of these 1D maps for all θ constitutes the 2D Radon transform of a yet unknown 2D density map (i.e., the desired 2D occupancy map). An inverse Radon transform is then applied to reconstruct the 2D density map. In addition, NLSA also retrieves the noise-reduced images at each point in this map. As in the 1D case, this procedure must next be performed for the eigenvector pairs corresponding to $\{\text{CM}_1, \text{CM}_2\}$ in all other Ω_{PD} embeddings.

F. Experimental Data

In the following, we provide a more thorough description of the two experimental data sets used.

80S Ribosome. This data set was collected by the Frank lab as a control in the course of a study of translational initiation by a plant virus, and then used in the first ManifoldEM analysis [15]. It consists of 849,914 cryo-EM images of the yeast 80S ribosome. Images were recorded with a 4k×4k Tietz CCD detector mounted on an FEI Tecnai Polara electron microscope, with complete TEM details available in the supplementary material of the original study [15]. The use of CCD detectors is a distinction worth noting, as more modern approaches typically rely on direct electron detectors for recording images. For our purposes, each projection, which was originally of dimension 250×250 with a pixel size of 1.5 Å, was downsampled to 125×125 with a pixel size of 3 Å. The effect of this downsampling on the geometry of the manifold embeddings is negligible, and as validated by a separate ground-truth analysis, insignificant for our analysis. Finally, we aligned the particles and obtained their orientations using the "non-uniform refinement" module in cryoSPARC [18]. The realigned (and downsampled) dataset of 80S ribosomes has been made available for download on the IEEE DataPort [19].

RyR1. This data set was collected by Ran Zalk *et al.* [20], and consists of 360,724 cryo-EM images of RyR1 prepared from rabbit skeletal muscle. It was subsequently used in the second ManifoldEM analysis [21] as the first of two RyR1 collections, referred to therein as the "ligand-free" collection. Images were recorded using an FEI Tecnai F30 Polara on a GATAN K2 Summit direct electron detector. Each projection has dimension 336×336 with a pixel size of 1.255 Å. The original papers should be referenced for a thorough account of details relating to the TEM and image processing [20, 21].

G. ESPER: Additional Details

In this section, additional details are provided for specific steps in the ESPER method, including additional pseudocode and intuition for procedures.

Subspace Partitioning. Here, the pseudocode for the *subspace partitioning* procedure is provided (i.e., Algorithm 3). Detailed notes on these procedures have been provided in our published repositories [3, 22], which includes comments describing less-significant decisions not explicitly noted here.

Algorithm 3: Subspace Partitioning.

Input: Rotated CM subspace, $\{\widehat{\Psi}_i \times \widehat{\Psi}_j\}$, and for each point, the corresponding image and CTF; least-squares fit, $\widehat{\Psi}_{\text{fit}}$.
Output: CM state assignments for each point (i.e., image) in $\{\widehat{\Psi}_i \times \widehat{\Psi}_j\}$; 1D occupancy map; 2D conformational movie.
Parameters: Maximum ball-tree distance, $\mathcal{B}_r$, and minimum number of points for keeping a ball-tree cluster, $\mathcal{B}_p$ for ball tree algorithm $\mathcal{B}(\mathcal{B}_r, \mathcal{B}_p)$ [23]; the alpha parameter, α: as increased, the point cloud will be fit with a tighter bounding box by alpha shapes algorithm $\mathcal{A}(\alpha)$ [24]; even number of CM states, δ.

scale both eigenvectors $\{\widehat{\Psi}_i \times \widehat{\Psi}_j\}$ between $[-1, 1]$
apply $\cos^{-1}$: $\{\widehat{\Psi}_i \times \widehat{\Psi}_j\} \to \{\widehat{\Phi}_i \times \widehat{\Phi}_j\}$; $\widehat{\Psi}_{\text{fit}} \to \widehat{\Phi}_{\text{fit}}$
apply $\mathcal{B}(\mathcal{B}_r, \mathcal{B}_p)$ to temporarily prune outlier points
split $\widehat{\Phi}_{\text{fit}}$ into halves $\widehat{\Phi}_{\text{fit}}^h$ via v_x in vertex $v \coloneqq \{v_x, v_y\}$
split $\{\widehat{\Phi}_i \times \widehat{\Phi}_j\}$ into halves $\{h_1, h_2\} \in \mathcal{H}$ via v_x
$\beta_S \coloneqq [\,] \in \beta$, $1 \le S \le \delta$; one β_S bin per CM state S
for h **in** $\mathcal{H}$ **do**
  apply $\mathcal{A}(\alpha)$ to enclose most of h with a polygon $\mathcal{P}_h$
  reinclude previous $\mathcal{B}(\mathcal{B}_r, \mathcal{B}_p)$ outliers in h
  $\{x, y\} \coloneqq \mathcal{P}_h \cap \widehat{\Phi}_{\text{fit}}^h$, defined at outer boundary of $\mathcal{P}_h$
  $\kappa \coloneqq \{v_x, y\}$; anchor point for ray projections
  for each 2D point p **in** h **do**
    $r_p \coloneqq$ ray connecting κ and p
    project p onto $\widehat{\Phi}_{\text{fit}}^h$ via $r_p \cap \widehat{\Phi}_{\text{fit}}^h$; index via $\widehat{\Phi}_{\text{fit}}^h\{p\}$
  $\Sigma \coloneqq [\,]$
  for $\theta \in [0°, 90°]$ via 1° increments **do**
    $r_\theta \coloneqq$ ray emanating from κ
    $\mathcal{P}_h^\theta \coloneqq$ lower sub-polygon formed by $r_\theta \cap \mathcal{P}_h$
    append area of $\mathcal{P}_h^\theta$ to Σ
  if h_1 **do** $S_h \coloneqq \{1, 2, \dots, \frac{\delta}{2}\}$ **else do** $S_h \coloneqq \{\frac{\delta}{2} + 1, \dots, \delta\}$
  split $\widehat{\Phi}_{\text{fit}}^h$ into $\frac{\delta}{2}$ equal-area segments S_h via Σ
  for $\widehat{\Phi}_{\text{fit}}^h\{p\}$ **in** each S_h **do** $\widehat{\Phi}_{\text{fit}}^h\{p\} \to \beta_{S_h}\{p\}$
for β_S **in** β **do**
  tally $\beta_S\{p\}$ for occupancy of state S
  CTF correct images corresponding to points $\beta_S\{p\}$
  add CTF-corrected images for frame S of 2D movie
return β_S image indices, 1D occupancy map, 2D movie

Conformation Compilation. In the following figure, we provide some basic intuition for our method of generating multidimensional free-energy landscapes and corresponding 3D movies using the ESPER "intersection of image-indices" approach within each PD manifold. For this schematic, we have simplified the problem from that of accounting for a parabolic surface (as observed throughout this study) to a plane; however, the following intuition remains the same in either case.

Just like the plane in Figure 17-A, PD images (represented by points) are organized along n orthogonal degrees of freedom (CMs) on a higher-dimensional hypersurface. For our needs,

this hypersurface can be approximated as a parabolic surface. Given the aforementioned uncertainty and difficulty in identifying and mapping this surface directly, we can instead refer to its set of n orthogonal projections (e.g., Figure 17-B and 10-C), which can be found and mapped with less difficulty. In the case of the plane, as in the case of our simplified illustration, these subspaces are 1D, while for a PD embedding, a 2D subspace is required to adequately capture each parabolic component. Recall that we identify these subspaces after performing eigenvector rotations to align the parabolic surface, such that only the CM parabola is visible in each of the respective 2D subspaces. Once located, we straighten each CM trajectory in each of these lower-dimensional projections into a 1D trajectory, such that the parabola is transformed into rectilinear form. Next, we partition the points separately into β contiguous bins (here, β = 20), and collect the set of image indices falling into each bin. Note that the size of the bin effectively defines the precision to which we can locate each point on the plane, and determines the range of images falling within each state for our final outputs.

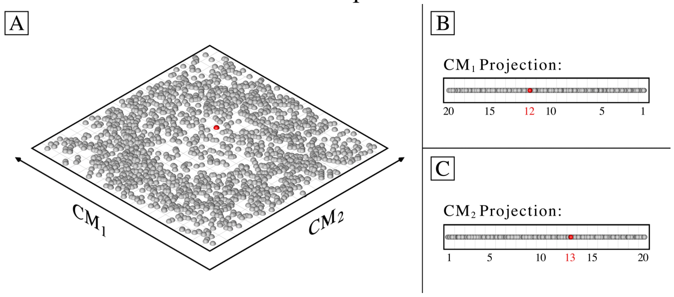

Fig. 17. A schematic to provide intuition for our intersection of image-indices approach, which simplifies the complex parabolic features in our observed PD embeddings into linear ones, on a plane. Let the red point near the center of the plane [A] represent an image with image index p_i, such that p_i belongs to both $CM_1\{12\}$ and $CM_2\{13\}$, and thus $p_i \in CM_1\{12\} \cap CM_2\{13\}$. This point, along with all others in the intersection $CM_1\{12\} \cap CM_2\{13\}$, is used to both define an occupancy and reconstruct a 3D density map for the respective state 12_13.

As a result, we are left with $n\beta$ sets of image indices combined across each set of CM coordinates for each PD. For ease of explanation in the following notation, assume $n = 2$. Next, we construct an empty $\beta \times \beta$ (i.e., β^n) array $\boldsymbol{P}$ and fill each element $P_{x,y}$ with the set of all image indices in the intersection $CM_1\{x\} \cap CM_2\{y\}$, where x and y are bins from CM_1 and CM_2, respectively. Since embeddings from each PD were obtained independently, we must also correct for *sense* (the directionality of the CM coordinates) as we accumulate indices in $\boldsymbol{P}$. At the end of this procedure, we sum the total number of entries in each $P_{x,y}$ to form a $\beta \times \beta$ occupancy map, which can then be converted into a free-energy landscape via the Boltzmann relation. We additionally use the indices of images within each $P_{x,y}$ to reconstruct a 3D density map for the set of corresponding images; in this example, producing 400 3D density maps in total. Naturally, this construction can be easily extended to three or more degrees of freedom.

Thus, given only a set of CM subspaces–each a parabolic trajectory defining an orthogonal degree of freedom–and with knowledge of the higher-dimensional relationship between them (i.e., the parabolic surface, as determined throughout our heuristic analysis), we can reconstruct that joint geometrical relationship using only the intersection of image indices obtained in all pairwise combinations of bins from straightened CM coordinates. In effect, this procedure only requires that we collect the indices of images, without the need to integrate them into 1D occupancy maps. This is in contrast to the previous ManifoldEM methodology employing NLSA, which discards the original image indices. This action carries a price, however, and must be reversed by performing a lengthy tomographic reconstruction using the 1D occupancy maps to obtain the 2D distribution, as described in Supplementary Material Section E.

H. Supplementary Movies

In the following, we provide a more thorough description of each supplementary movie referenced throughout the text.

Movie 1. An animation cycling through the 400 SS_2 states as represented by the Hsp90 atomic structures (top) and corresponding PDs (bottom). The ordering of these states chronologically follows the indices described using our second-hand and minute-hand analogy. Specifically, for SS_2, the ordering of the states of CM_2 progresses like the second hand of a clock, with each progression from $CM_2\{1\} \rightarrow CM_2\{20\}$ iterating CM_1 (akin to the minute hand) forward once. Thus, of the 400 states in SS_2, the first index corresponds to state $CM_1\{1\}\_CM_2\{1\}$, the second to state $CM_1\{1\}\_CM_2\{2\}$, the 21st to state $CM_1\{2\}\_CM_2\{1\}$, and the 400th to state $CM_1\{20\}\_CM_2\{20\}$.

Movie 2. Visualization of mouth-wing construction and continuum. In the first scene, on the right is a set of animations cycling through the 400 SS_2 states of the mouth-wing toy model, as viewed from four example PDs. Of these, the northeast PD was used for the investigation in Figure 5. On the left is a static render of the toy model (state 400) as it is rotated 360°. The next scene of Movie 2 showcases the sequence of operations performed to generate the mouth-wings model in Cinema 4D. In the last scene, projections of the final model show it animating along a conformational trajectory representing the diagonal of the state space.

Movie 3. Effect of applying a 4D orthogonal rotation to the 4D subspace—shown here using four 2D projections of that subspace—obtained from applying PCA on PD_2 in data-type II (SS_2 with SNR of 0.1 and $\tau = 10$). The six rotation matrices required for the rotation of a 4D subspace are shown on the right. As can be seen, by only applying rotation operator $R_{2,3}$ with 28.65°, both CM_1 and CM_2 parabola-housing subspaces are corrected—preserving all distances between points—such that they reside completely in the plane of $\{PC_1, PC_3\}$ and $\{PC_2, PC_4\}$, respectively.

Movie 4. An example presentation of the 2D histogram approach for finding optimal angles of rotation for realigning CM eigenfunctions. Here, the effect of an incremental 4D rotation operator $R_{2,3}$ on each 2D subspace is shown. During these rotations, each 2D subspace exhibits a unique profile

which can be characterized by the number of nonzero bins in the corresponding 2D histogram as a function of angle.

Movie 5. An example PD from data-type II is chosen to demonstrate the inner workings of our eigenfunction realignment algorithm. Here, a $d = 5$ dimensional subspace is first isolated, with each 2D subspace therein assigned an $\mathcal{R}^2$ value based on least-square fits. Given presence of adequate fits, the parabola-housing subspace in each eigenvector row is determined via the best $\mathcal{R}^2$ value, with the corresponding eigenvector indices used to procure the four rotational operators (of 10, for $d = 5$) required to align each point cloud with the plane of its subspace. We next demonstrate the generation of 2D histograms as these operators are exercised to determine the optimal angles. Although slight inaccuracies may emerge during the histogram optimization, these are typically no more than 5°, and prove insignificant for downstream procedures.

Movie 6. Set of 2D movies captured along SS_2 subspaces, with each corresponding Ω_{PD} generated from images with SNR of 0.1 and $\tau = 5$. As seen in both the first and second row, as a result of the integration procedure on each corresponding CM parabola, there is a significant difference in resolution between the desired CM and the CM orthogonal to it. When 3D movies are eventually constructed from the full set of these 2D movies on S^2, pairwise information from both CMs is incorporated into each volume such that these anomalies resolve.

Movie 7. Set of 2D movies produced by ESPER from data-type IV in SS_2, corresponding to five PDs equispaced on a great circle. Unlike in Movie 6, which does not incorporate CTF, here the set of CTF-corrected and Wiener-filtered snapshots within each bin are integrated. Overall, the resolution of desired CM content in each row is superb across all states.

Movie 8. Set of 3D reconstructions produced by ESPER from data-type IV in SS_2, corresponding to 126 PDs, SNR of 0.1 and CTF. A sequence of 69 3D density maps is shown, as seen from four orthographic views animated along a chosen trajectory in the retrieved 2D state space. Here, the 2D occupancy map before $\mathcal{R}^2$ thresholding has been supplied (in contrast to Figure 9). All 3D density maps were reconstructed using the RELION [25] "relion_reconstruct" module, without removal of any images in the original ensemble. Post-processing steps for display included removal of dust (via the Chimera [26] "hideDust" command with size 10) and application of a Gaussian filter (via Chimera, using 1 Å standard deviations of the 3D isotropic Gaussian function).

Movie 9. For our comparison of ESPER and NLSA, three example PDs were chosen from data-type IV for direct comparison of their 2D movies and corresponding occupancy maps. In the first segment of Movie 9, PD_2 represents the class of extremely well-behaved embeddings, having near-perfect eigenfunction pre-alignment and irrelevant inward curling at the boundaries for both CM_1 and CM_2 point clouds. Next, as discussed in the main text, PD_{33} is a representative from the class of embeddings with eigenfunctions that are appreciably unaligned from the ideal eigenbasis. Finally, PD_{49} required a minimal d-dimensional rotation (much like PD_2), but exhibited significant inward curling at the boundaries of its subspaces. The last segment of Movie 9 demonstrates the final 2D movies (one per eigenvector) output by NLSA for PD_{33} and PD_{49}.

Movie 10. A comparison of the results from ESPER and NLSA as performed on the same Ω_{PD} embedding of the 80S ribosome data set. This embedding was chosen as an example specifically for the presence of a leading subspace with "robust" parabolic geometry, as obtained from a PD with 1825 images defined within a 2° angular width. At the end of Movie 10, a schematic is provided detailing the location of ribosomal domains as observed from the current viewing direction. We note that the caption "PNAS" is used only to match the CMs we obtained in this study using the Python ManifoldEM suite [27] with those described in the original study [15].

Movie 11. A comparison of the results from ESPER and NLSA as performed on the same Ω_{PD} embedding from the RyR1 data set. Although only housing 976 images within a 6° angular width, this PD was chosen as an example due to the presence of a noticeable—albeit "marginal"—parabolic appearance of a leading subspace (CM_1). Overall, however, the occupancy of PDs with smaller angular widths was insufficient for producing embeddings with robust geometric features. For the 2D movies, the ESPER method was run with $\delta = 10$ bins, with the first three eigenvectors of the SVD decomposition retained for noise filtering. We note that the caption "Nat Commun" is used only to match the CMs we obtained in this study using the Python ManifoldEM suite [27] with those described in the original study [21].

I. Glossary of Terms

Term	Definition
**80S ribosome**	The ribosome, the molecular machine for protein synthesis in eukaryotes
**alignment file**	A file that contains values of geometric parameters (such as shifts, Eulerian angles) and microscope parameters (such as defocus) for each image. Such a file, in standard format, is used by many cryo-EM image processing packages
**atomic description of state**	The full set of coordinates of all atoms of a molecule that is in that state
**contrast transfer function (CTF)**	The function (of spatial frequency) describing the linear relationship between the Fourier transform of the object projection and the Fourier transform of the observed image intensity. This description is valid for weak phase objects and a transmission electron microscope operated in the brightfield mode
**Coulomb potential map**	3D electrostatic potential distribution; the result of 3D reconstruction from projections in cryo-EM
**CTF double-filtering kernel**	A kernel used in Euclidean distance calculations, introduced in [15], that renders two images obtained with different CTFs virtually equivalent. Without such a kernel, the manifold mapping would pick up differences in imaging parameters, which have no relevance in the CM ordering of image content
**Fourier shell correlation (FSC)**	A function (of spatial frequency) used to measure reproducibility of the results of a 3D reconstruction from projections. Projection data are randomly split in half, and the resulting reconstructions are compared over shells in Fourier space. The FSC falls off with increasing spatial frequency, and the point where it falls below a given threshold nominally marks the resolution
**ligands**	Small molecules that bind to, or detach from, a macromolecule or molecular machine in the course of biological function
**microscopy parameters**	Values of parameters that define the imaging and electron optical settings of the microscope, and thereby the CTF, such as electron wavelength, spherical aberration constant, defocus, and magnification
**molecular machine**	A macromolecule that performs a work cyclic in the cell. It is driven by energy from the thermal environment and GTP or ATP hydrolysis, and often involves binding and release of ligand molecules
**RyR1**	Ryanodine receptor, or calcium release channel. A large molecule in skeletal muscle responsible for nerve excitation-triggered muscle contraction through calcium release
**SARS-CoV2 spike protein**	The protein, at the periphery of the SARS-CoV-2 virus causing the pandemic COVID-19 disease, that is making contact with a receptor of the human host, a step which is instrumental for the process of infection

Supplementary References

[1] E. Seitz *et al.*, "Simulation of cryo-EM ensembles from atomic models of molecules exhibiting continuous conformations," *bioRxiv*, 2019, doi: 10.1101/864116
[2] E. Seitz, "Cryo-EM synthetic continua repository," Zenodo, 2019. Available: https://github.com/evanseitz/cryoEM_synthetic_continua, doi: 10.5281/zenodo.3561105.
[3] E. Seitz, "ManifoldEM : ESPER data and code repository," IEEE Dataport, 2022. doi: https://dx.doi.org/10.21227/wmn8-k054
[4] Schrodinger, LLC, "The PyMOL molecular graphics system," 2015.
[5] G. Tang *et al.*, "EMAN2: An extensible image processing suite for electron microscopy," *J Struct Biol*, vol. 157, no. 1, pp. 38–46, 2007.
[6] J. Frank, *Three-Dimensional Electron Microscopy of Macromolecular Assemblies: Visualization of Biological Molecules in Their Native State*. Oxford, New York: Oxford University Press, 2006.
[7] Maxon Computer GmbH. Cinema4D. Version 24, 1989–2022.
[8] Baxter *et al.*, "Determination of signal-to-noise ratios and spectral SNRs in cryo-EM low-dose imaging of molecules", J Struct Biol, vol. 166, no. 2, pp. 126–32, 2009.
[9] N. Fischer *et al.*, "Ribosome dynamics and tRNA movement by time-resolved electron cryomicroscopy," *Nature*, vol. 466, no. 7304, pp. 329–33, 2010.
[10] P. Gilbert, "The reconstruction of a three-dimensional structure from projections and its applications to electron microscopy.", *Proc R Soc Lond B Biol Sci*, vol. 182, no. 1066, pp. 89–102, 1972.
[11] R. Coifman and S. Lafon, "Diffusion maps," *Appl Comput Harmon A*, vol. 21, no. 1, pp. 5–30, 2006.
[12] Z. Li *et al.*, "A comparative study of different distances for similarity estimation", *Intelligent Computing and Information Science*, pp. 483–8, Springer Berlin Heidelberg, 2011.
[13] A. Ferguson *et al.*, "Systematic determination of order parameters for chain dynamics using diffusion maps", Proc Natl Acad Sci USA, vol. 107, no. 31, pp. 13597–602, 2010.
[14] R. Coifman *et al.*, "Graph Laplacian tomography from unknown random projections", *IEEE Trans Image Process*, vol. 17, no. 10, pp. 1891–9, 2008.
[15] A. Dashti *et al.*, "Trajectories of the ribosome as a Brownian nanomachine," *P Natl A Sci*, vol. 111, no. 49, pp. 17492–97, 2014.
[16] D. Lay *et al.*, *Linear Algebra and its Applications*. Pearson, ed. 5, 2016.
[17] D. Giannakis and A. Majda, "Nonlinear Laplacian spectral analysis for time series with intermittency and low-frequency variability," *Proc Natl Acad Sci USA*, vol. 109, no. 7, pp. 2222–7, 2012.
[18] A. Punjani *et al.*, "Non-uniform refinement: Adaptive regularization improves single-particle cryo-EM reconstruction", *Nature Methods*, vol. 17, pp. 1214–21, 2020.
[19] J. Pallesen, G. Sharma, A. Meskauskas, V. Stupina, A. Simon, J. Dinman, J. Frank, "Cryo-EM dataset of 80S ribosomes from yeast," IEEE Dataport, 2022, doi: https://dx.doi.org/10.21227/cq30-ak65
[20] R. Zalk *et al.*, "Structure of a mammalian ryanodine receptor", *Nature*, vol. 517, no. 7532, pp. 44–9, 2015.
[21] A. Dashti *et al.*, Retrieving functional pathways of biomolecules from single-particle snapshots," *Nat Commun*, vol. 11, no. 1, p. 4734, 2020.
[22] E. Seitz, "ManifoldEM: ESPER repository," Zenodo, 2021. Available: https://github.com/evanseitz/cryoEM_ESPER, 10.5281/zenodo.5362645.
[23] F. Pedregosa *et al.*, "Scikit-learn: Machine learning in Python," *Journal of Machine Learning Research*, vol. 12, no. 85, pp. 2825–30, 2011.
[24] K. Bellock *et al.*, "Python alpha shape repository," Zenodo, 2021, doi: 10.5281/zenodo.4603211
[25] S. Scheres. "RELION: Implementation of a Bayesian approach to cryo-EM structure determination", *J Struct Biol*, vol. 180, no. 3, pp. 519–30, 2012.
[26] E. Pettersen *et al*. "UCSF Chimera–a visualization system for exploratory research and analysis", *J Comput Chem*, vol. 25, no. 13, pp. 1605–12, 2004.
[27] E. Seitz *et al.*, "ManifoldEM Python repository," Zenodo, 2021. Available: https://github.com/evanseitz/ManifoldEM_Python, doi: 10.5281/zenodo.5578874
